# Cognitive correlates of decision-making in Parkinson’s disease: a systematic review

**DOI:** 10.3389/fnagi.2026.1840023

**Published:** 2026-06-12

**Authors:** Johanna Büchel, Marc Montgomery Lässer, Noelia Peña Arauzo, Katharina Thaler, Jens Carsten Möller, Alessandra Fanciulli, Philipp Mahlknecht, Laura Zamarian

**Affiliations:** 1 Private University in the Principality of Liechtenstein (UFL), Triesen, Liechtenstein; 2 Department of Neuropsychology, Rehabilitation Clinic Walzenhausen, Clinic Group Valens, Walzenhausen, Switzerland; 3 Department of Neurology, Medical University Innsbruck, Innsbruck, Austria; 4 Division of Neurorehabilitation, Fribourg Hospital, Meyriez-Murten, Switzerland

**Keywords:** associations, gambling, methodological variability, neuropsychological testing, real-life decision-making

## Abstract

**Introduction:**

Changes in decision-making (DM) are frequently observed in patients with Parkinson’s disease (PD). Despite extensive research, the relationships between DM and various cognitive functions remain incompletely understood. This systematic review aims to synthesize existing evidence on the link between DM and cognition in PD, emphasizing a comprehensive perspective that considers multiple cognitive domains and various DM tasks.

**Methods:**

A systematic literature search was conducted across PubMed, PubPsych, and LIVIVO databases. Inclusion criteria encompassed studies that examined the relationship between DM performance and cognitive functions in individuals with PD. Data extraction focused on sample characteristics, types of DM tasks employed, cognitive domains assessed, and key findings on their association.

**Results:**

A total of 38 studies, published between 2001 and 2025, involving 1,313 individuals with PD, were identified. Most of these studies used laboratory-based DM tasks—with the Iowa Gambling Task (IGT) as the most frequently used DM paradigm, followed by the Game of Dice Task (GDT)—whereas only few studies employed ecologically valid DM tasks that simulate real-life DM contexts. A substantial number of studies investigated the association of DM performance with global cognitive status, executive functions, and memory. Fewer studies explored social cognition, psychomotor speed and attention, visuo-construction and visuo-spatial skills, language, and numerical abilities. Most analyses on the association between DM and cognition in PD revealed non-significant findings. Among the studies reporting significant associations, findings did not consistently cluster around specific cognitive domains or DM tasks, indicating heterogeneity in results.

**Conclusion:**

This systematic review highlights the complex and often inconsistent relationship between cognitive functions and DM in individuals with PD. Despite some evidence of significant associations, most findings are non-significant, scattered, and influenced by methodological variability across studies. Future research should aim for standardized, ecologically valid assessments and consider the multifaceted nature of cognition and DM to better elucidate these relationships in PD.

## 1 Introduction

Parkinson’s disease (PD) is a neurodegenerative disorder primarily affecting motor functions, with the cardinal symptoms of bradykinesia, rigidity, tremor, and postural instability. Beyond these characteristic motor symptoms, individuals with PD frequently experience a range of non-motor symptoms, including autonomic dysfunction, sleep disorders, and cognitive impairments (Seppi et al., 2019; Fanciulli et al., 2026). In PD, cognition can range from normal to dementia, with cognitive impairments often emerging early in the disease course, even before the onset of motor symptoms in the prodromal phase (Aarsland et al., 2017; Peña Arauzo et al., 2025). Cognitive deficits commonly affect executive functions, attention, visuospatial skills, and memory. Recent evidence also suggests impairments in decision-making (DM).

DM is a complex cognitive process that involves multiple steps, including evaluating and weighing different options, anticipating potential gains and losses, framing situations, learning from feedback, and processing outcomes (Brand et al., 2006; Ernst and Paulus, 2005; Trepel et al., 2005). It interacts closely with attention, executive functions, memory, and emotional processing, reflecting its reliance on a complex network of brain regions and neurotransmitter systems (Rangel et al., 2008; Ryterska et al., 2013). Neuroimaging and neuropsychological studies have identified several key structures involved in DM, such as the amygdala, orbitofrontal cortex, ventromedial prefrontal cortex, anterior cingulate cortex, dorsolateral prefrontal cortex, parietal cortex, as well as ventral and dorsal striatum (Ryterska et al., 2013). These regions contribute to different aspects of DM, with their involvement varying depending on the decision context and the level of uncertainty involved (Hsu et al., 2005; Schultz et al., 2008). A substantial body of work highlights the critical role of the basal ganglia in DM, with deficits observed in conditions such as PD, where these structures are compromised (Ryterska et al., 2013). Alterations in DM processes are evident both with normal aging and more prominently in individuals with neurological or psychiatric conditions. Effective DM is crucial for social, health-related, and financial outcomes; impairments can lead to negative consequences that affect individuals’ current and future wellbeing. Consequently, understanding DM deficits and their relationship to other cognitive functions, particularly in patients with brain lesions, is of great relevance.

Research on DM in PD has yielded highly heterogeneous findings. Some studies report that PD patients exhibit poorer performance compared to healthy controls, while others find no significant differences. Several investigations employing the Iowa Gambling Task (IGT) (Bechara et al., 1994), a laboratory-based paradigm that assesses DM under initial ambiguity, indicate that PD patients tend to be more risk-taking (even when they might understand that this is not the optimal behavior), make more disadvantageous choices, and have greater difficulty learning from feedback than healthy controls (Ryterska et al., 2013; Colautti et al., 2021; Evens et al., 2016; Poletti et al., 2011; Sinz et al., 2008). However, not all studies have observed significant differences between PD patients and controls in IGT performance (Dirnberger and Jahanshahi, 2013; Perry and Kramer, 2015), highlighting the variability in results across the literature. Studies investigating DM under risky conditions have mostly used the Game of Dice Task (GDT) (Brand et al., 2004), demonstrating impairments in PD patients in comparison to healthy controls (Brand et al., 2004; Euteneuer et al., 2009). Besides these predominantly used laboratory-based gambling tasks, DM performance in PD patients has also been investigated through tasks that more closely mimic everyday DM situations. Here, too, there is considerable variability in the findings, with some studies reporting significant differences from healthy controls (Rosen et al., 2015), while others do not (Ryterska et al., 2013). As noted in some previous reviews (Ryterska et al., 2013; Sinz et al., 2008; Colautti et al., 2023), these highly heterogeneous findings are likely due to methodological issues, including variations in sample sizes and type, cognitive profiles, medication state, and task focus and characteristics. According to Ryterska et al. (2014), DM deficits among PD patients do not reflect a global or generalized impairment. Instead, they stem from impairments in specific sub-components of the DM process—namely, the valuation or cost–benefit analysis stage and the outcome evaluation stage—which are likely more vulnerable in individuals with PD than other DM sub-components (e.g., the representation stage, which involves constructing a representation of the decision problem/scenario). In a meta-analysis of 38 studies, including a total of 60 separate experiments of DM across different tasks (Ryterska et al., 2013), PD patients were found to be impaired relative to healthy controls in 65% of all experiments.

Numerous explanations for DM deficits in PD have been proposed, ranging from neuropathological changes in cortical and subcortical networks involved in DM (Ryterska et al., 2013; Colautti et al., 2021; Poletti et al., 2011; Gleichgerrcht et al., 2010), to dopamine dysregulation caused by the disease itself or medication (Evens et al., 2016; Gleichgerrcht et al., 2010; Cools et al., 2001; Vaillancourt et al., 2013), to a variety of psychiatric symptoms that may influence DM performance, including depression, anxiety, apathy, and impulse control disorders (ICDs) (Colautti et al., 2021; Martini et al., 2018; Voon et al., 2017), as well as alterations in cognitive and emotional processes involved in DM (Colautti et al., 2021; Colautti et al., 2023; Gleichgerrcht et al., 2010). While deficits in executive functions have been primarily emphasized as playing a role in DM under risky conditions, alterations in emotional processing have typically been suggested in relation to DM under ambiguous conditions (Colautti et al., 2023). Although existing review articles provide a useful foundation on the link between DM performance and cognition in PD, they have notable limitations. Only one review (Colautti et al., 2023) specifically focusses on this relationship; others mention cognition only as a secondary or possible explanatory factor (Colautti et al., 2021; Poletti et al., 2011; Sinz et al., 2008). Additionally, these review articles tend to focus narrowly on common laboratory-based DM tasks, primarily the IGT, and mainly emphasize executive functions, with other cognitive domains receiving little attention. They also reference a limited number of original studies (between 6 and 13) on cognition and DM in PD, indicating a somewhat restricted scope of existing research coverage.

This systematic review aims to synthesize existing evidence on the relationship between DM and cognition in PD. By moving beyond the analysis of individual DM tasks or specific cognitive domains, it strives to present a broader, more integrated perspective on how DM performance relates to different cognitive functions. This approach considers that individuals with PD often face complex decisions in various contexts with varying demands, involving multiple steps of the DM process, and that several cognitive functions may contribute to performance. Through this approach, this review aims to identify potential patterns and underlying relationships between DM performance and cognitive functions among individuals with PD.

## 2 Methods

This systematic review was conducted in accordance with the Preferred Reporting Items for Systematic Reviews and Meta-Analyses (PRISMA) guidelines (Page et al., 2021). The study was not preregistered.

### 2.1 Search strategy

We conducted a comprehensive literature search in PubMed (Medline), PubPsych, and LIVIVO for articles available until December 2025. While other databases (e.g., PsycInfo, Web of Science, PSYNDEX) were considered, they were ultimately excluded due to a lack of open search access, redundancy with the selected databases, and limited anticipated added value. The sensitive search strategy was built around three key concepts: PD, DM, and cognition. For each concept, a broad range of keywords and synonyms were identified. These included among others: “Parkinson’s Disease” OR “Parkinson”; AND “decision making” OR “deciding”; AND “cognition” OR “cognitive functions” OR “cognitive abilities” OR “cognitive deficits” OR “neuropsychology” (a detailed description of the literature search and evaluation process is made available on request). These terms were then combined into a single search string for each database using Boolean operators and were adapted to leverage database-specific search techniques such as wildcards and phrase searching. Although possible Medical Subject Headings (MeSH) were initially identified, pilot searches showed that their inclusion significantly reduced search specificity, resulting in a high number of irrelevant results. Therefore, we employed a keyword-based strategy to increase precision and ensure the retrieval of studies that explicitly referenced our constructs of interest. Furthermore, we manually screened the reference lists of relevant review or meta-analysis articles for additional studies. The Rayyan QCRI program (Ouzzani et al., 2016) was used to manage the search results, check for duplicates, and screen for eligibility.

Two researchers (JB and LZ) defined the selection procedure. The literature search was conducted by one researcher (JB), who prepared a list of articles for screening. Two independent reviewers (JB and MML) then screened the titles and abstracts against the predefined inclusion and exclusion criteria (see below). The full texts of potentially relevant articles were then reviewed for final decision. Any disagreements were resolved through discussion between the reviewers or by consultation with additional researchers (LZ and KT).

### 2.2 Inclusion and exclusion criteria

The predefined selection criteria were as follows:

*Inclusion criteria:* (1) peer-reviewed original research articles, (2) published in English or in German, (3) inclusion of PD patients who actively made decisions between options, (4) objective assessment of DM performance using a validated task or performance-based questionnaire, (5) objective assessment of at least one cognitive domain (global cognitive status, psychomotor speed and attention, memory, executive functions, visuo-construction and visuo-spatial skills, language, social cognition, or numerical skills) through a neuropsychological task, psychometric test, or diagnostic tool, and (6) explicit investigation of the relationship between DM and cognitive performance in PD using statistical analysis (correlation or regression-based analysis).

*Exclusion criteria:* (1) animal research, basic science studies, or mathematical modelling studies, (2) studies that explicitly included other specific forms of parkinsonism than idiopathic PD (e.g., atypical and secondary parkinsonism), (3) reliance exclusively on self-reported measures for DM or cognitive performance without objective assessments, (4) DM involving individuals other than the PD patients (e.g., caregivers), (5) “decision” tasks assessing the mere selection or identification of the correct option (e.g., lexical decision tasks) or targeting learning processes rather than DM itself (e.g., probabilistic learning tasks), and (6) the cognitive evaluation was embedded within the DM task itself (e.g., feedback processing, stimulus–reward learning) without an independent neuropsychological measure.

In summary, this review examined “classical” cognitive variables—such as attention, executive functions, and memory—routinely assessed in neuropsychology, without restricting the scope to specific domains. Processes like reward processing, feedback learning, and rule learning, when integral to the DM tasks themselves, were not considered as separate cognitive variables. Regarding DM, this review focused on tasks involving the evaluation of at least two alternatives, each with distinct probabilities, benefits, risks, and outcomes, where the individual needs to make a decision that requires some form of deliberation based on the available information. The focus is on the DM process or the decision itself. Therefore, this review excluded tasks that do not involve this evaluative, strategic process, such as simple response selection tasks (e.g., Go/NoGo, visual discrimination) or learning tasks where decisions become automatic or are based solely on learned associations, even when these were referred to as DM tasks by the authors.

### 2.3 Data extraction and key measurements

Data extraction was performed independently by three researchers (JB, MML, and NPA), with disagreements resolved by consensus or by a third reviewer (LZ). The extracted data included: sample type and size, demographical and clinical characteristics, the DM task and type evaluated, cognitive assessment tools and domains, and outcomes from correlation and regression-based analyses. When information could not be confidently inferred from the text, the study’s authors were contacted via email. The most frequent issues concerned: (1) which specific cognitive variables were included in correlation or regression analyses, and (2) the participant subgroups over which these analyses were performed. If clarification was unavailable, we applied the following rules: For issue (1), if a restriction was indicated (e.g., selective discussion of certain cognitive variables), only the explicitly mentioned results were recorded; and if no restriction was apparent (e.g., “all cognitive variables”), we assumed the statement applied to the full set of cognitive variables. For issue (2), a footnote under the Supplementary Tables S1-S5 reported in Supplementary material indicates the studies where this uncertainty occurred.

### 2.4 Quality assessment and risk of bias

The methodological quality and risk of bias of the included studies were evaluated using the JBI critical appraisal checklist for analytical cross-sectional studies (Barker et al., 2026). This assessment was conducted by three independent reviewers (JB, MML, and NPA). Disagreements were resolved through discussion. To enhance the specificity and relevance of the evaluation concerning the review objectives, three of the eight questions were explicitly tailored to focus on the constructs of interest: Question 3 addressing cognition, Question 4 concerning DM, and Question 7 regarding the association between these variables (for detailed information, see Supplementary material).

### 2.5 Statistical analysis

We evaluated the proportion of articles that examined the association between DM and global cognition or specific cognitive domains in PD patients, noting whether significant results were reported. Based on these findings, we identified the DM tasks for which inferences about relationships with multiple cognitive domains can be made. An additional meta-analysis was not performed because either the number of available results for a specific association was insufficient or, despite an adequate number of studies, the measures were too heterogeneous to combine them meaningfully.

## 3 Results

### 3.1 Study selection

**Figure 1:**
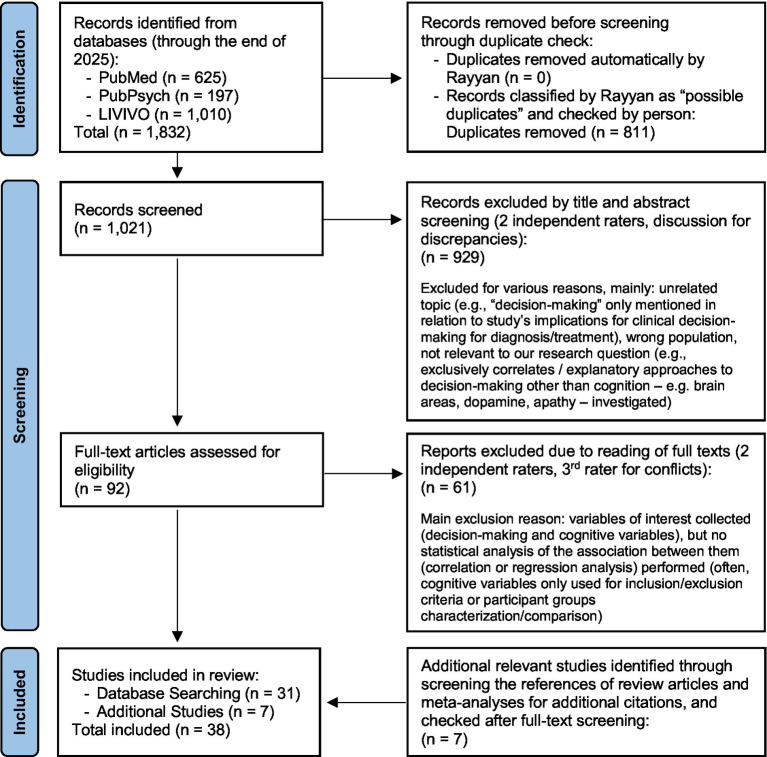
Flow diagram of the study selection process. The flow diagram summarizes the identification, screening, eligibility assessment, and inclusion of studies.

Initial database searches conducted in June 2023 identified 1,488 records. An updated search in April 2026 to identify more recent studies published between 2023 and December 2025 added a further 344 records, resulting in a total of 1,832 potentially relevant records. After removing duplicates and screening titles and abstracts, 92 studies underwent full-text review, of which 31 studies met all inclusion and none of the exclusion criteria. A further 7 relevant records were identified by screening the reference lists of review articles and meta-analyses. Thus, a total of 38 studies, published from 2001 to 2025, were included in the systematic review (Figure 1).

### 3.2 Characteristics of the included studies

Table 1 reports the PD samples’ demographic and clinical characteristics of the 38 studies included. Total sample sizes ranged from 14 to 97 participants (median: 25), with a total of 1,313 individuals with PD.

**Table 1:** Demographic and clinical characteristics of the PD samples of the included studies.

Study	Population	N PD total	N PD per subgroup	% male^a^	Ø/Mdn age (y)^a^	Ø/Mdn education (y)^a,b^	Ø/Mdn disease duration (y)^a^	H&Y (Ø/Mdn/range)^a^	Ø/Mdn LEDD (mg)^a^	Medication state (ON/OFF)
Amstutz et al. (2025)	PD perioperative STN-DBS (pre- and early postoperative; about half with ICD)	43	10	69.8	60.2	n.a.	12.3	n.a.	651.0	ON
	PD with chronic STN-DBS (about half with ICD)		33							
Arshad et al. (2017)	RPD	20	10	50.0	67.0	n.a.	7.9	1.4	n.a.	n.a.
	LPD		10							
Boller et al. (2014)	PD with bilateral STN-DBS	18		50.0	64.3	n.a.	13.6	n.a.	519.6	ON + OFF
Brand et al. (2004)	PD	20		55.0	66.9	9.1	8.8	3.0	n.a.	n.a.
Brandt et al. (2015)	PD with bilateral STN-DBS	30	15	50.0	66.0	16.1	n.a.	≤ 3	1307.0	n.a.
	PD without bilateral STN-DBS		15							
Buelow et al. (2014)	PD with apathy	24	10	54.2	68.0	15.6	n.a.	2.4	468.4^c^	n.a.
	PD without apathy		14							
Caballero et al. (2022)	PD	15		53.3	70.3	16.5	n.a.	1 to 3	n.a.	n.a.
Colautti et al. (2024)	PD	42		52.4	66.4	9.9	5.7	n.a.	492.0	ON
Czernecki et al. (2002)	PD	23		39.1	57.6	11.5	14.9	on: 2.2 off: 3.8	1115.3^c^	ON + OFF
Danesin et al. (2022)	PD	62		62.9	68.5	11.7	n.a.	n.a.	n.a.	n.a.
Delazer et al. (2009)	PD without dementia	39	20	59.0	70.3	11.1	6.6	2.1	617.1^c^	ON
	PD with dementia		19							
de Rezende Costa et al. (2016)	De-novo, untreated PD	25		68.0	54.2	14.0	1.4	1.9	-	-
Djamshidian et al. (2012)	PD without ICB	53	27	86.8	62.1	14.0	10.5	n.a.	835.3	ON
	PD with ICB		26							
Dymek et al. (2001)	PD with cognitive impairment	20		n.a.	75.0	14.3	n.a.	n.a.	n.a.	n.a.
Euteneuer et al. (2009)	PD	21		33.3	67.6	11.1	7.1	2.5	487.7	n.a.
Eygelshoven et al. (2017)	PD	46		65.2	61.0	n.a.	5.2	2.1	564.7	ON
Gescheidt et al. (2012)	Early-onset PD (≤ 45 y)	19		73.7	50.3	n.a.	11.3	1.7	1259.0	ON
Ibarretxe-Bilbao et al. (2009)	PD	24		66.7	56.1	11.0	3.1	1.7	299.6	n.a.
Kobayakawa et al. (2008)	PD	34		35.3	69.9	13.2	6.4	1.5	391.0	ON
Kobayakawa et al. (2010)	PD	14		50.0	68.9	14.8	5.6	1.4	476.9	ON
Kobayakawa et al. (2017)	PD	20		50.0	70.1	13.5	6.3	1.9	383.4	n.a.
Kobayashi et al. (2019)	PD without ICD	25	15	60.0	63.0	12.3	7.5	2.8	556.6	ON + OFF
	PD with ICD		10							
Marques et al. (2022)	PD with RBD	60	40	60.0	62.9	12.1	7.4	2.0	749.2	n.a.
	PD without RBD		20							
Martínez-Horta et al. (2013)	PD with apathy	37	20	n.a.	66.8	n.a.	6.1	2.0	614.9^c^	ON
	PD without apathy		17							
Martini et al. (2018)	PD with ICD	25	13	84.0	64.0	13.7	7.7	2.5	504.2	ON
	PD without ICD		12							
Mimura et al. (2006)	PD	18		27.8	68.9	n.a.	n.a.	2 to 3	n.a.	n.a.
Oyama et al. (2011)	PD with bilateral STN-DBS^d^	32	16	34.4	64.9	n.a.	11.1	n.a.	770.5^c^	ON
	PD without DBS		16							
Pagonabarraga et al. (2007)	PD	35		62.9	67.2	11.9	8.4	2.2	870.0	ON
Perretta et al. (2005)	Early PD (= H&Y 1–2.5)	32	16	53.1	75.1	14.4	n.a.	2.7	n.a.	ON
	Later PD (= H&Y 3–4)		16							
Poletti et al. (2010)	De-novo, untreated PD	30		73.3	64.9	9.3	0.9	n.a.	-	-
Rosen et al. (2013)	PD	19		36.8	65.2	10.3	5.8	2.5	854.4	ON
Rosen et al. (2015)	PD	20		70.0	67.5	13.5	8.4	2.5	999.4	ON
Sáez-Francàs et al. (2014)	PD with fatigue	89	33	65.2	62.3	15.2	4.5	1.9	517.9	ON
	PD without fatigue		56							
Scott et al. (2025)	PD with motivational disturbance (apathy or ICDs)	97	54	66.0	67.2	15.8	n.a.	1 to 3	742.8^c^	n.a.
	PD without motivational disturbance		43							
Stout et al. (2001)	PD	22		59.1	66.0	14.2	7.7	2.6	n.a.	n.a.
Ueda et al. (2022)	PD	59		47.5	65.9	n.a.	9.7	2 to 4	877.0	ON
Xi et al. (2015)	PD	15		46.7	60.7	8.9	4.3	2.0	254.6	n.a.
Zapf et al. (2022)	PD	86		62.8	66.5	13.5	6.3	2.0	613.8	ON
Total		1,313								
Mdn		25		57.0	66.2	13.5	7.3	2.1^e^	614.4	
Mean^f^		34.6		56.5	65.3	12.8	7.4	2.2^e^	671.2	
SD^f^		21.1		13.8	5.1	2.2	3.2	0.5^e^	268.4	
Min		14		27.8	50.3	8.9	0.9	1.4^e^	254.6	
Max		97		86.8	75.1	16.5	14.9	3.0^e^	1307.0	

^a^For studies reporting only subgroup data, study-level means were calculated as weighted averages based on subgroup sizes.

^b^The variable “Education (years)” was generally reported without specifying whether vocational training was included. The higher mean values (mostly >11 years) likely reflect the inclusion of vocational training. Four studies reported lower values (<10 years): two explicitly indicated they only included school or formal education (Brand et al., 2004; Poletti et al., 2010), while in two studies values were unspecified (Colautti et al., 2024; Xi et al., 2015). Whether or not these four studies are included does not affect the median or maximum values. However, excluding them results in changes to the minimum value (from 8.9 to 10.3), the mean value (from 12.8 to 13.4), and the standard deviation (from 2.2 to 1.8).

^c^These values should be interpreted with some caution, as in these cases, the LEDD (levodopa equivalent daily dose) was not explicitly reported. Instead, either only the LED was mentioned (without specifying “daily”), or only the daily levodopa dose was reported (which was used as a proxy for LEDD when no additional dopaminergic medications were reported; however, unreported additional dopaminergic medications cannot be excluded, potentially leading to an underestimate of LEDD). Alternatively, both the levodopa daily dose and the LEDD of dopamine agonists were reported separately. In such cases, an approximate LEDD was calculated by summing up these values, provided no other medications were indicated. As a consequence, these values can be considered equivalent or close estimates of the actual LEDD.

^d^Although cognition and decision-making were assessed pre- and post-operatively in the DBS-group, the correlation analyses with cognitive variables were based exclusively on the IGT scores obtained durin the pre-session. Therefore, for the analysis of interest in this review, the total sample of the study still did not have DBS.

^e^For studies that reported only a range, an approximate mean was calculated by taking the midpoint of the reported range.

^f^Means and standard deviations were calculated to describe study-level characteristics without weighting by sample size.

Ø, Mean; H&Y, Hoehn and Yahr Scale; ICB, Impulsive-compulsive behavior; ICD, Impulse control disorder; LEDD, levodopa equivalent daily dose; LPD, PD with predominantly left-sided motor symptoms; Max, Maximum; Mdn, Median; Min, Minimum; N, number; n.a., not available/not specified; PD, Parkinson’s Disease; RBD, REM (Rapid eye movement) sleep behavior disorder; RPD, PD with predominantly right-sided motor symptoms; SD, Standard deviation; STN-DBS, Subthalamic Nucleus Deep Brain Stimulation; y, years.

The included studies comprised PD participants with a median age of 66.2 years and a median of 13.5 years of education. There was a median of 57% male participants per study. The median disease duration was 7.3 years, whereas the disease severity, as measured by the Hoehn & Yahr scale, covered the full spectrum from mild to severe stages with a median of 2.1. The medication dose was typically reported as levodopa equivalent daily dose (LEDD) with a median of 614.4 mg.

As no other specific forms were explicitly indicated, all studies likely included patients with idiopathic PD. These samples were generally characterized by the absence of significant comorbidities. Common exclusion criteria of the included studies comprised other neurological or psychiatric diseases (e.g., history of stroke, ICDs, or depression), substance abuse/dependence, pathological gambling, or implanted deep brain stimulation (DBS). Furthermore, most studies employed cognitive screening tools with predefined cut-off scores to exclude participants with dementia or clinically relevant cognitive impairment. Participants were generally on parkinsonian medication. When medication state during assessment was reported, patients were typically tested in the ON state, occasionally as part of an ON vs. OFF comparison. Data on the side of onset and on PD subtype (i.e., tremor-dominant, akinetic-rigid, or other classifications) were rarely and inconsistently reported across studies (and therefore not indicated in Table 1). Deviating from this common profile, some studies focused systematically on specific PD subgroups: de-novo, untreated PD patients (de Rezende Costa et al., 2016; Poletti et al., 2010), DBS patients (Amstutz et al., 2025; Boller et al., 2014; Brandt et al., 2015), early-onset PD patients (Gescheidt et al., 2012), PD patients with (overall mild) cognitive impairment (Dymek et al., 2001), with apathy (Buelow et al., 2014; Martínez-Horta et al., 2013), with fatigue (Sáez-Francàs et al., 2014), with ICDs (Martini et al., 2018; Kobayashi et al., 2019), with motivational disturbance (i.e., apathy and/or ICD) (Scott et al., 2025), with impulsive-compulsive behaviors (Djamshidian et al., 2012), with dementia (Delazer et al., 2009), or with rapid eye movement (REM) sleep behavior disorder (Marques et al., 2022).

### 3.3 Quality assessment and risk of bias

The appraisal results are summarized in Supplementary Table S7 and Supplementary Figure S2. Overall, the quality of the studies ranged from moderate to high. All studies (100%) employed valid and reliable measures for DM (Q4), and nearly all (93.8%) used valid and reliable tools to measure cognition (Q3). A clear description of the inclusion and exclusion criteria (Q1) was given in 25 studies (78.1%), while 24 studies (63.2%) explicitly reported the use of objective, standardized diagnostic criteria for PD (Q2). Only 22 studies (57.9%) offered detailed descriptions of the study sample and setting (Q8). Regarding confounding factors, 35 studies (92.1%) identified potential confounders (Q5), but only 24 (63.2%) explicitly stated that they used strategies to deal with them (Q6). The weakest aspect emerging from the critical appraisal related to statistical analysis (Q7), with only five studies (13.2%) being evaluated positively. Although all studies employed appropriate statistical methods to examine the association between DM and cognition, several limitations were noted. These included small sample sizes without *a priori* power analyses (leading to underpowered studies and less precise estimates) and a lack of correction for multiple testing despite conducting numerous analyses, which increased the risk of Type I error. Additionally, some analyses regarded pooled samples of PD patients and healthy controls, limiting the validity and interpretability of PD-related inferences. Overall, no study was excluded based on the outcomes of the critical appraisal. However, the methodological limitations, particularly regarding the association analyses and the description of the setting (especially the lack of reporting on whether PD patients were assessed during their medication ON or OFF state), should be taken into consideration when discussing the findings.

### 3.4 Assessment of DM

Our analysis reveals a predominant focus on laboratory-based paradigms over social or applied DM contexts (for details, Supplementary Tables S1–S6). Laboratory-based paradigms included the IGT (Bechara et al., 1994), the Deal or No Deal task (Post et al., 2008), the Balloon Analogue Risk Task (BART) (Lejuez et al., 2002), the GDT (Brand et al., 2004), the Probability-Associated Gambling Task (PAG) (Zamarian et al., 2008), the Economic Choice task (Kobayashi et al., 2019), the Beads task (Furl and Averbeck, 2011), the Kirby Delayed Discounting Questionnaire (Kirby et al., 1999), the Framing Paradigm (Kahneman and Tversky, 1984; Tversky and Kahneman, 1986), the cognitive adaptation of the Effort Expenditure for Rewards Task (COG-EEfRT) (Scott et al., 2025), and the Apple Tree Task (Chong et al., 2015). Social DM was assessed through the Dictator Game (Kahneman et al., 2019; Morishima et al., 2012), a calculation-based social DM task (Niederle and Vesterlund, 2007), the Trust Game (Berg et al., 1995), and the Everyday Moral DM task original and revised (Starcke et al., 2011). Applied DM paradigms included the Capacity to Consent to Treatment Instrument (CCTI) (Marson et al., 1995), the MacArthur Competence Assessment Tool for Treatment (MacCAT-T) (Grisso et al., 1997), and the financial judgments subtest of the Numerical Activities of Daily Living—Financial (NADL-F) short battery (Toffano et al., 2021).

Laboratory-based paradigms, such as the IGT, Deal or No Deal task, BART, GDT, PAG task, and Economic Choice task, simulate fictitious gambling scenarios. In these paradigms, the primary objective is to maximize a hypothetical monetary outcome by choosing between at least two options with varying probabilities of gain or loss. Tasks like the IGT, Deal or No Deal task, and BART involve DM under ambiguity, where outcome probabilities and rules are unknown or implicit and must be learned through feedback. Conversely, paradigms such as the GDT, PAG task, and Economic Choice task involve DM under risk, where probabilities and outcomes are explicitly stated or calculable. Additional laboratory-based paradigms focus on specific facets of DM, including information sampling before making a choice (the Beads task), delayed discounting (assessing choices between smaller immediate rewards and larger delayed rewards; the Kirby Delayed Discounting Questionnaire), the impact of information framing on choices (the Framing Paradigm), and effort-based DM (weighing the required physical effort, as in the Apple Tree Task, or cognitive effort, as in the COG-EEfRT, against the potential reward). Social DM paradigms, such as the Dictator Game, the calculation-based social DM task, the Trust Game, and the Everyday Moral DM task, assess DM in socially contextualized situations, focusing on aspects such as altruism/egoism, fairness, trust, or moral reasoning, and the adjustment of behavior or decisions depending on social cues or fictitious counterparts. Finally, applied DM tasks measure functional DM competence in “real-life like” scenarios, addressing everyday topics such as medical decisions (e.g., CCTI, MacCAT-T) or financial decisions (e.g., NADL-F). These tasks often require integrating the available information to reach a decision or a value-based judgment and, in some cases, assess individual decisional steps. It is important to note that the distinction between laboratory-based, social, and applied DM paradigms is not mutually exclusive, and is based on the primary focus and contextual characteristics of the different DM paradigms.

In summary, the IGT was used in 21 studies (55.3%) to assess DM under initial ambiguity, either administered alone or in combination with other tasks (Euteneuer et al., 2009; Buelow et al., 2014; Colautti et al., 2024; Czernecki et al., 2002; Delazer et al., 2009; Gescheidt et al., 2012; Ibarretxe-Bilbao et al., 2009; Kobayakawa et al., 2008; Kobayakawa et al., 2010; Kobayakawa et al., 2017; Marques et al., 2022; Mimura et al., 2006; Oyama et al., 2011; Pagonabarraga et al., 2007; Perretta et al., 2005; Poletti et al., 2010; Sáez-Francàs et al., 2014; Stout et al., 2001; Ueda et al., 2022; Xi et al., 2015). Other studies assessing DM under ambiguity adopted the Deal or No Deal task (Brandt et al., 2015) or the BART (Martini et al., 2018; Buelow et al., 2014). For assessing DM under risk, the most frequently used instrument was the GDT (*n* = 6 studies, 15.8%) (Brand et al., 2004; Euteneuer et al., 2009; Boller et al., 2014; Brandt et al., 2015; Colautti et al., 2024; Xi et al., 2015). Other studies on DM under risk adopted the PAG task (Delazer et al., 2009) or the Economic Choice task (Kobayashi et al., 2019). The remaining studies using laboratory-based paradigms focused on the following aspects: information sampling (Beads task) (de Rezende Costa et al., 2016; Djamshidian et al., 2012), delayed discounting (Kirby Delayed Discounting Questionnaire) (Martini et al., 2018), framing effects (Framing Paradigm) (Brandt et al., 2015), or effort-based DM with either cognitive (COG-EEfRT) (Scott et al., 2025) or physical effort components (Apple Tree Task) (Amstutz et al., 2025). Social and applied DM contexts were assessed by a few studies (*n* = 5, 13.2%, and *n* = 3, 7.9%, respectively), using highly heterogeneous tasks (Rosen et al., 2015; Arshad et al., 2017; Caballero et al., 2022; Danesin et al., 2022; Dymek et al., 2001; Eygelshoven et al., 2017; Rosen et al., 2013; Zapf et al., 2022). This reflects the inclusion of studies employing a diverse array of DM tasks, covering different facets and paradigms. For a summary of the number of studies using a specific DM task, see Supplementary Figure S1 (panel A).

### 3.5 Assessment of cognition

The most frequently investigated associations with DM in PD patients involved executive functions (*n* = 32 studies, 84.2%) (Brand et al., 2004; Euteneuer et al., 2009; Rosen et al., 2015; Martini et al., 2018; Amstutz et al., 2025; Boller et al., 2014; Caballero et al., 2022; Colautti et al., 2024; Czernecki et al., 2002; Delazer et al., 2009; de Rezende Costa et al., 2016; Djamshidian et al., 2012; Dymek et al., 2001; Eygelshoven et al., 2017; Gescheidt et al., 2012; Ibarretxe-Bilbao et al., 2009; Kobayakawa et al., 2008; Kobayakawa et al., 2010; Kobayakawa et al., 2017; Kobayashi et al., 2019; Martínez-Horta et al., 2013; Mimura et al., 2006; Oyama et al., 2011; Pagonabarraga et al., 2007; Perretta et al., 2005; Poletti et al., 2010; Rosen et al., 2013; Sáez-Francàs et al., 2014; Stout et al., 2001; Ueda et al., 2022; Xi et al., 2015; Zapf et al., 2022), global cognitive status (*n* = 23 studies, 60.5%) (Brand et al., 2004; Euteneuer et al., 2009; Amstutz et al., 2025; Boller et al., 2014; Brandt et al., 2015; Buelow et al., 2014; Czernecki et al., 2002; Kobayakawa et al., 2008; Kobayakawa et al., 2010; Kobayakawa et al., 2017; Kobayashi et al., 2019; Marques et al., 2022; Martínez-Horta et al., 2013; Mimura et al., 2006; Oyama et al., 2011; Pagonabarraga et al., 2007; Perretta et al., 2005; Poletti et al., 2010; Scott et al., 2025; Stout et al., 2001; Ueda et al., 2022; Xi et al., 2015; Zapf et al., 2022), and memory (*n* = 13 studies, 34.2%) (Brand et al., 2004; Martini et al., 2018; Colautti et al., 2024; Czernecki et al., 2002; Dymek et al., 2001; Eygelshoven et al., 2017; Kobayakawa et al., 2010; Kobayakawa et al., 2017; Kobayashi et al., 2019; Martínez-Horta et al., 2013; Pagonabarraga et al., 2007; Stout et al., 2001; Xi et al., 2015). Conversely, only a limited number of studies focused on other cognitive domains such as social cognition (*n* = 9, 23.7%) (Euteneuer et al., 2009; Rosen et al., 2015; Caballero et al., 2022; Ibarretxe-Bilbao et al., 2009; Mimura et al., 2006; Rosen et al., 2013; Ueda et al., 2022; Xi et al., 2015; Zapf et al., 2022), psychomotor speed and attention (*n* = 8, 21.1%) (Martini et al., 2018; Amstutz et al., 2025; Dymek et al., 2001; Eygelshoven et al., 2017; Ibarretxe-Bilbao et al., 2009; Martínez-Horta et al., 2013; Sáez-Francàs et al., 2014; Stout et al., 2001), visuo-construction and visuo-spatial skills (*n* = 7, 18.4%) (Brand et al., 2004; Dymek et al., 2001; Kobayakawa et al., 2010; Kobayakawa et al., 2017; Martínez-Horta et al., 2013; Stout et al., 2001; Xi et al., 2015), language (*n* = 2, 5.3%) (Dymek et al., 2001; Martínez-Horta et al., 2013), and numerical skills (*n* = 2, 5.3%) (Arshad et al., 2017; Danesin et al., 2022). As detailed in Tables 2, 3, there was considerable heterogeneity in the measures used to assess the different cognitive domains, often targeting various subdomains. For a summary of the number of studies assessing a specific cognitive domain, see Supplementary Figure S1 (panel B).

**Table 2:** Summary of DM tasks and cognitive measures separated by study and cognitive domain (global cognitive status, psychomotor speed/attention, memory, visuoconstruction/visuospatial skills).

Study	DM task	Global cognitive status	Psychomotor speed/attention	Memory	Visuoconstruction/visuospatial skills
Amstutz et al. (2025)	**Apple Tree Task**	MoCA	TMT-A, **Symbol search (WAIS-IV)**	–	–
Arshad et al. (2017)	Dictator Game	–	–	–	–
Boller et al. (2014)	GDT	MDRS, PANDA, MMSE	–	–	–
Brand et al. (2004)	GDT	MMSE, DemTect	–	Word list (DemTect)	Visuospatial rotation (LPS-7)
Brandt et al. (2015)	DND, **GDT**, Framing	**MoCA**	–	–	–
Buelow et al. (2014)	IGT, BART	MMSE, MoCA	–	–	–
Caballero et al. (2022)	Trust Game	–	–	–	–
Colautti et al. (2024)	IGT, GDT	–	–	Digit span forward	–
Czernecki et al. (2002)	IGT	MDRS	–	Grober free recall	–
Danesin et al. (2022)	Financial judgments (NADL-F)	–	–	–	–
Delazer et al. (2009)	IGT, PAG	–	–	–	–
de Rezende Costa et al. (2016)	Beads	–	–	–	–
Djamshidian et al. (2012)	Beads	–	–	–	–
Dymek et al. (2001) ^c^	**CCTI**	–	TMT-A, **Attention (DRS)**, Mental control (WMS-R), Digit span forward + backward total	**Memory (DRS)**, Logical memory I (WMS-R), **Logical memory II (WMS-R)**	Construction (DRS)
Euteneuer et al. (2009)	IGT, GDT	MMSE, DemTect	–	–	–
Eygelshoven et al. (2017)	**MacCAT-T**	–	**Digit Trial (CST)**, Word card (Stroop), Letter-Digit Substitution Test	Digit span forward, Rivermead Behavioral Memory Test, Verbal Learning Test	–
Gescheidt et al. (2012)	IGT	–	–	–	–
Ibarretxe-Bilbao et al. (2009)	IGT	–	Sustained Attention (CPT II)	–	–
Kobayakawa et al. (2008)	IGT	MMSE	–	–	–
Kobayakawa et al. (2010)	**IGT**	MMSE	–	**Digit span forward**^a^, Figural delayed recall (ROCFT)	Figure copy (ROCFT)
Kobayakawa et al. (2017)	IGT	MMSE	–	Figural delayed recall (ROCFT)	Figure copy (ROCFT)
Kobayashi et al. (2019)	Economic Choice	MMSE	–	Figural delayed recall (ROCFT)	–
Marques et al. (2022)	IGT	MMSE	–	–	–
Martínez-Horta et al. (2013)	IGT	PD-CRS	Sustained attention (PD-CRS)	Verbal memory (PD-CRS)	Posterior-Cortical score (PD-CRS), Clock copy (PD-CRS)
Martini et al. (2018)	BART, **Kirby Delayed Discounting**	–	**Verbal generation (Hayling-1)**, TMT-A, Divided attention (TAP)	**Composite memory score (Logical memory WMS + Memory CAMCOG)**	–
Mimura et al. (2006)	IGT	MMSE	–	–	–
Oyama et al. (2011)	IGT	MMSE	–	–	–
Pagonabarraga et al. (2007)	**IGT**	MMSE, **MDRS**	–	**Verbal delayed free recall (RAVLT)**	–
Perretta et al. (2005)	IGT	MMSE	–	–	–
Poletti et al. (2010)	IGT	MMSE	–	–	–
Rosen et al. (2013)	Everyday Moral DM	–	–	–	–
Rosen et al. (2015)	Everyday Moral DM	–	–	–	–
Sáez-Francàs et al. (2014)	**IGT**	–	Word reading (Stroop-1), **Colour naming (Stroop-2)**^b^, TMT-A	–	–
Scott et al. (2025)	**COG-EEfRT**	**MoCA**	–	–	–
Stout et al. (2001)	IGT	MDRS	Attention (MDRS)	Memory (MDRS)	Construction (MDRS)
Ueda et al. (2022)	IGT	MMSE	–	–	–
Xi et al. (2015)	IGT, **GDT**	MMSE	–	Digit span forward, **Verbal delayed free recall (RAVLT)**	Hooper Visual Organisation Task
Zapf et al. (2022)	Calculation-based social DM	PANDA	–	–	–

Only the DM and cognitive tasks assessed in the original study that met our inclusion criteria and were included in correlation or regression analyses are listed in this table. To enhance clarity, the table presents only the tasks incorporated into statistical analyses without detailing the specific outcome variables assessed. It is important to note that, across the studies, different outcome variables were employed for certain tasks (particularly the IGT). Entries highlighted in bold indicate a significant association (either in bivariate analyses or within multivariable models) between the DM task and the (sub-)test assessing a specific cognitive domain.

^a^This correlation was found significant for the modified version of the IGT but not for the original version.

^b^This correlation was found significant for non-fatigued PD patients but not for fatigued PD patients.

^c^For each CCTI standard, only the four strongest correlations with cognitive variables were reported. As such, it remains unclear whether the remaining cognitive variables exhibited non-significant correlations or whether significant but weaker correlations were found. Therefore, in the context of the results of this study, it cannot be ruled out that cognitive tasks currently marked as non-significant (not bolded) may, in fact, have yielded significant associations. Notably, all significant results pertain specifically to the CCTI standards S1, S4, and/or S5.

BART, Balloon Analogue Risk Task; CAMCOG, Cambridge Cognition Examination; CCTI, Capacity to Consent to Treatment Instrument; COG-EEfRT, cognitive adaptation of the Effort Expenditure for Rewards Task; CPT II, Conners’ Continuous Performance Test II; CST, Concept Shifting Test; DemTect, Demenz-Detektion; DM, Decision-Making; DND, Deal or no Deal; GDT, Game of Dice Task; IGT, Iowa Gambling Task; LPS, Leistungsprüfsystem; MacCAT-T, MacArthur Competence Assessment Tool for Treatment; (M)DRS, (Mattis) Dementia Rating Scale; MMSE, Mini Mental State Examination; MoCA, Montreal Cognitive Assessment; NADL-F, Numerical Activities of Daily Living – Financial (short battery); PAG, Probability-Associated Gambling Task; PANDA, Parkinson Neuropsychometric Dementia Assessment; PD-CRS, Parkinson’s Disease-Cognitive Rating Scale; RAVLT, Rey Auditory Verbal Learning Test; ROCFT, Rey-Osterrieth Complex Figure Test; TAP, Testbatterie zur Aufmerksamkeitsprüfung; TMT, Trail Making Test; WAIS-IV, Wechsler Adult Intelligence Scale - Fourth Edition; WMS(-R), Wechsler Memory Scale (-revised).

**Table 3:** Summary of DM tasks and cognitive measures separated by study and cognitive domain (executive functions, language, numerical skills, social cognition).

Study	DM task	Executive functions	Language	Numerical skills	Social cognition
Amstutz et al. (2025)	Apple Tree Task	Interference inhibition (Stroop)	–	–	–
Arshad et al. (2017)	**Dictator Game**	–	–	**Number pair bisection task**	–
Boller et al. (2014)	GDT	Phonemic verbal fluency, Logical thinking (LPS-4)	–	–	–
Brand et al. (2004)	**GDT**	Semantic and phonemic verbal fluency, Logical thinking (LPS-4), **MCST**, Digit span backward	–	–	–
Brandt et al. (2015)	DND, GDT, Framing	–	–	–	–
Buelow et al. (2014)	IGT, BART	–	–	–	–
Caballero et al. (2022)	Trust Game	Semantic verbal fluency	–	–	Faux-Pas Test
Colautti et al. (2024)	**IGT** ^**a**^ **, GDT** ^**b**^	Semantic, **phonemic**^**a**^ and **alternate**^**a**^ **verbal fluency, verbal fluency shifting index**^**a**^, **Interference inhibition (Stroop)**^**a,b**^**, Digit span backward**^**a**^	–	–	–
Czernecki et al. (2002)	**IGT**	**Frontal score**, Semantic and phonemic verbal fluency, MCST	–	–	–
Danesin et al. (2022)	**Financial judgments (NADL-F)**	–	–	**Counting currencies, Item purchase**, Reading abilities, Bill payments, Percentages, Financial concepts (NADL-F)	–
Delazer et al. (2009)	**IGT** ^**c**^ **, PAG** ^**d**^	Semantic verbal fluency, Go-NoGo (FAB), Digit span backward, **OMO**^d^, **TMT-B**^c^	–	–	–
de Rezende Costa et al. (2016)	Beads	Go-NoGo (FAB)	–	–	–
Djamshidian et al. (2012)	Beads	Working memory task	–	–	–
Dymek et al. (2001) ^e^	**CCTI**	**EXIT-25**, Initiation/Perseveration (DRS), Semantic and phonemic verbal fluency, **Comprehension (WAIS-R)**, Similarities (WAIS-R), Conceptualization (DRS), **TMT-B**	Boston Naming Test, Token test, Simple auditory comprehension test	–	–
Euteneuer et al. (2009)	IGT, **GDT**	Semantic and **phonemic verbal fluency**, Logical thinking (LPS-4), **MCST**, Digit span backward	–	–	Reading the Mind in the Eyes Test
Eygelshoven et al. (2017)	MacCAT-T	Phonemic verbal fluency, Shift and Digit Trial divided (CST), Interference inhibition (Stroop), Digit span backward	–	–	–
Gescheidt et al. (2012)	IGT	Tower of London, Interference inhibition (Stroop)	–	–	–
Ibarretxe-Bilbao et al. (2009)	**IGT**	**Digit span backward**	–	–	**Ekman test**
Kobayakawa et al. (2008)	IGT	WCST	–	–	–
Kobayakawa et al. (2010)	IGT	WCST, Digit span backward	–	–	–
Kobayakawa et al. (2017)	IGT	FAB, WCST	–	–	–
Kobayashi et al. (2019)	Economic Choice	FAB	–	–	–
Marques et al. (2022)	IGT	–	–	–	–
Martínez-Horta et al. (2013)	IGT	Fronto-Subcortical score (PD-CRS), Clock drawing (PD-CRS), Working memory (PD-CRS), Alternating verbal fluency (PD-CRS), Action verbal fluency (PD-CRS)	Confrontation naming (PD-CRS)	–	–
Martini et al. (2018)	**BART**, Kirby Delayed Discounting	Executive function (CAMCOG), TMT-B, Rule detection (BSAT), **Go-NoGo (TAP)**, Verbal inhibition (Hayling-2)	–	–	–
Mimura et al. (2006)	**IGT**	Maze-tracing (WISC-R), semantic and phonemic verbal fluency, WCST, Interference inhibition (Stroop)	–	–	**Reading the Mind in the Eyes Test**
Oyama et al. (2011)	IGT	FAB, KWCST	–	–	–
Pagonabarraga et al. (2007)	**IGT**	**Semantic and phonemic verbal fluency**, Interference inhibition (Stroop), Digit span backward	–	–	–
Perretta et al. (2005)	IGT	WCST, Stroop (1 + 2)	–	–	–
Poletti et al. (2010)	IGT	FAB	–	–	–
Rosen et al. (2013)	Everyday Moral DM	Semantic and phonemic verbal fluency, MCST	–	–	Reading the Mind in the Eyes Test
Rosen et al. (2015)	Everyday Moral DM	Key Search Test (BADS), Logical thinking (LPS-4), TMT B-A, MCST	–	–	Reading the Mind in the Eyes Test
Sáez-Francàs et al. (2014)	IGT	Tower of London, TMT B-A, Interference inhibition (Stroop-3, Stroop-4)	–	–	–
Scott et al. (2025)	COG-EEfRT	–	–	–	–
Stout et al. (2001)	IGT	Initiation/Perseveration (MDRS), Conceptualization (MDRS)	–	–	–
Ueda et al. (2022)	**IGT**	FAB, TMT B-A, **MCST**, Interference inhibition (Stroop)	–	–	Facial Emotion Recognition Test
Xi et al. (2015)	**IGT**, GDT	Semantic verbal fluency, Interference inhibition (Stroop), Digit span backward	–	–	**Reading the Mind in the Eyes Test**
Zapf et al. (2022)	Calculation-based social DM	TMT B/A, MCST	–	–	Reading the Mind in the Eyes Test

Only the DM and cognitive tasks assessed in the original study that met our inclusion criteria and were included in correlation or regression analyses are listed in this table. To enhance clarity, the table presents only the tasks incorporated into statistical analyses without detailing the specific outcome variables assessed. It is important to note that, across the studies, different outcome variables were employed for certain tasks (particularly the IGT). Entries highlighted in bold indicate a significant association (either in bivariate analyses or within multivariable models) between the DM task and the (sub)test assessing a specific cognitive domain.

^a^These correlations were found significant for the IGT.

^b^This correlation was found significant for the GDT.

^c^There was a significant correlation between IGT performance and TMT-B for PD patients without dementia, but not for PD patients with dementia.

^d^There was a significant correlation between PAG performance and OMO for PD patients without dementia, but not for PD patients with dementia.

^e^For each CCTI standard, only the four strongest correlations with cognitive variables were reported. As such, it remains unclear whether the remaining cognitive variables exhibited non-significant correlations or whether significant but weaker correlations were found. Therefore, in the context of the results of this study, it cannot be ruled out that cognitive tasks currently marked as non-significant (not bolded) may, in fact, have yielded significant associations. Notably, all significant results pertain specifically to the CCTI standards S1, S4, and/or S5.

BADS, Behavioural Assessment of the Dysexecutive Syndrome; BART, Balloon Analogue Risk Task; BSAT, Brixton Spatial Anticipation Test; CAMCOG, Cambridge Cognition Examination; CCTI, Capacity to Consent to Treatment Instrument; COG-EEfRT, cognitive adaptation of the Effort Expenditure for Rewards Task; CST, Concept Shifting Test; DM, Decision-Making; DND, Deal or no Deal; EXIT-25, Executive Interview; FAB, Frontal Assessment Battery; GDT, Game of Dice Task; IGT, Iowa Gambling Task; (K)WCST, (Keio) Wisconsin Card Sorting Test; LPS, Leistungsprüfsystem; MacCAT-T, MacArthur Competence Assessment Tool for Treatment; MCST, Modified Card Sorting Test; (M)DRS, (Mattis) Dementia Rating Scale; NADL-F, Numerical Activities of Daily Living - Financial (short battery); NAI, Nürnberger Altersinventar; OMO, Odd Man Out; PAG, Probability-Associated Gambling Task; PD-CRS, Parkinson’s Disease-Cognitive Rating Scale; TAP, Testbatterie zur Aufmerksamkeitsprüfung; TMT, Trail Making Test; WAIS(-R), Wechsler Adult Intelligence Scale (-revised); WISC-R, Wechsler Intelligence Scale for Children-revised.

### 3.6 Associations between DM and cognition in PD

Tables 2 and 3 summarize the results on the investigated associations between DM and cognitive performance in PD patients. For clarity, we have not specified the direction of the significant associations in this section; instead, detailed information on these associations, including the statistical analysis performed, test statistics, their direction, and the sample type, is provided in Supplementary Tables S1–S5. Supplementary Table S6 provides a summary of the number of studies investigating specific DM-cognition combinations.

In general, there were no significant associations between performance on any of the DM tasks and measures of visuo-construction and visuo-spatial skills, or language abilities (Brand et al., 2004; Dymek et al., 2001; Kobayakawa et al., 2010; Kobayakawa et al., 2017; Martínez-Horta et al., 2013; Stout et al., 2001; Xi et al., 2015). In the following sections, we summarize the results separately for each of the other cognitive domains.

#### 3.6.1 Associations between DM and global cognitive status

Contrary to expectations, a higher global cognitive status, as measured by the Mattis Dementia Rating Scale (MDRS) total score, was associated with poorer performance on the IGT in an analysis conducted exclusively on PD patients (Pagonabarraga et al., 2007). Conversely, a higher Montreal Cognitive Assessment (MoCA) score was associated with better performance on the GDT in an analysis comprising PD patients with and without DBS, as well as healthy controls (Brandt et al., 2015). The analysis conducted for COG-EEfRT showed that higher global cognitive status, as indicated by the MoCA score, was associated with a greater frequency of hard task selections in trials with high reward probability conditions in a sample comprising PD patients with and without motivational disturbances (i.e., apathy and/or ICD) (Scott et al., 2025).

No significant associations were found between global cognitive status and other DM tasks (for details, Table 2).

#### 3.6.2 Associations between DM and psychomotor speed/attention

In non-fatigued PD patients, a significant correlation was observed between performance on the IGT and a Stroop subtest that requires participants to name the color of various symbols within a specified time limit (Sáez-Francàs et al., 2014). However, this correlation was not evident among PD patients experiencing fatigue (Sáez-Francàs et al., 2014). Additionally, the total score on the Kirby Delayed Discounting Questionnaire was found to correlate with scores on a Hayling subtest that assesses verbal generation during sentence completion (Martini et al., 2018). This analysis included PD patients with and without ICD, as well as healthy controls (Martini et al., 2018). Performance on the Symbol Search task served as a significant positive predictor of faster decision speed in the Apple Tree Task within a model with multiple predictor variables (Amstutz et al., 2025). This finding emerged from a combined sample of PD patients with recent DBS, including both pre- and postoperative assessments, as well as those with chronic DBS, with ICD present in approximately half of both subgroups (Amstutz et al., 2025). Further, in PD patients with overall mild cognitive impairment, the attention sub-score of the Dementia Rating Scale (DRS) was correlated with performance on two legal standards of the CCTI (Dymek et al., 2001). Supporting this, Eygelshoven et al. (2017) reported that performance on the Digit Trial of the Concept Shifting Test predicted medical DM as assessed by the MacCAT-T. In general, higher DM performance tended to be associated with better attention scores.

It is important to note that some measures showed no significant associations. Specifically, correlations between performance on the BART and attention measures did not reach statistical significance in a pooled sample of PD patients (with and without ICD) and healthy controls (Martini et al., 2018). Moreover, no significant associations were found, in general, between DM performance and measures of sustained or divided attention (Martini et al., 2018; Ibarretxe-Bilbao et al., 2009; Martínez-Horta et al., 2013) (see Table 2).

#### 3.6.3 Associations between DM and memory

Several measures were employed across studies to evaluate various aspects of memory. Kobayakawa et al. (2010) identified a significant correlation between short-term memory—specifically, the digit span forward—and performance on a modified version of the IGT. Notably, they did not find significant correlations for performance on the original IGT with memory measures (Kobayakawa et al., 2010). A significant correlation with measures of long-term verbal memory was observed both for the IGT (Pagonabarraga et al., 2007) and the GDT (Xi et al., 2015). Martini et al. (2018) reported a significant correlation between performance on the Kirby Delayed Discounting Questionnaire and a composite memory score in a pooled sample comprising PD patients—with and without ICD—and healthy controls. Correlations with different memory measures were also found for the CCTI (Dymek et al., 2001) (for details, Table 2). It is important to note that results on the relationship between memory and DM were heterogeneous: in some studies (Martini et al., 2018; Dymek et al., 2001; Xi et al., 2015), higher memory scores were associated with more advantageous DM performance, whereas others (Kobayakawa et al., 2010; Pagonabarraga et al., 2007) reported the opposite pattern.

No significant associations with memory were found for the Economic Choice task, BART, and MacCAT-T (Martini et al., 2018; Eygelshoven et al., 2017; Kobayashi et al., 2019). In general, there were no significant associations between DM tasks and visual long-term memory measures (see Table 2).

#### 3.6.4 Associations between DM and executive functions

The domain of executive functions was the most frequently investigated in relation to DM and PD (Table 3). Several studies reported significant correlations between performance on the IGT and diverse executive function measures. Specifically, IGT performance correlated significantly with a composite frontal score (Czernecki et al., 2002), verbal fluency (Colautti et al., 2024; Pagonabarraga et al., 2007), interference inhibition (Colautti et al., 2024), working memory (Colautti et al., 2024; Delazer et al., 2009; Ibarretxe-Bilbao et al., 2009), and measures of set-shifting and cognitive flexibility (Delazer et al., 2009; Ueda et al., 2022). Notably, a correlation between cognitive flexibility and IGT performance was observed in PD patients without dementia but not in those with dementia (Delazer et al., 2009). Martini et al. (2018) identified a correlation between inhibition and performance on the BART across a pooled sample comprising PD patients with and without ICD and healthy controls. Performance on the GDT also correlated with phonemic verbal fluency (Euteneuer et al., 2009), interference inhibition (Colautti et al., 2024), and measures of set-shifting and cognitive flexibility (Brand et al., 2004; Euteneuer et al., 2009). In a study on the PAG task, Delazer et al. (2009) found an association between DM performance and a measure of set-shifting and cognitive flexibility in non-demented PD patients but not in those with dementia. Furthermore, Dymek et al. (2001) reported that various standards of the CCTI correlated with different executive function measures, including a composite executive function score, cognitive flexibility, and judgement. Findings regarding the relationship between executive functions and DM were also heterogeneous: Some studies reported that higher executive functioning was associated with more favourable DM performance (Brand et al., 2004; Euteneuer et al., 2009; Martini et al., 2018; Colautti et al., 2024; Delazer et al., 2009; Dymek et al., 2001; Ibarretxe-Bilbao et al., 2009), whereas others (Colautti et al., 2024; Czernecki et al., 2002; Pagonabarraga et al., 2007; Ueda et al., 2022) found the opposite pattern.

No significant associations with executive functions were found for the Beads task, Economic Choice Task, Kirby Delayed Discounting Questionnaire, Apple Tree Task, calculation-based social DM task, Trust Game, Everyday Moral DM task, and MacCAT-T (Rosen et al., 2015; Martini et al., 2018; Amstutz et al., 2025; Caballero et al., 2022; de Rezende Costa et al., 2016; Djamshidian et al., 2012; Eygelshoven et al., 2017; Kobayashi et al., 2019; Rosen et al., 2013; Zapf et al., 2022). Additionally, no significant relationship between planning abilities and DM performance—independent of the DM task—was identified (see Table 3).

#### 3.6.5 Associations between DM and numerical skills

The relationships between DM performance and numerical skills have been examined in only two studies. Arshad et al. (2017) found a significant association between errors in a number pair bisection task and the amount of money donated during the Dictator Game. Similarly, Danesin et al. (2022) found significant associations in PD patients between a financial judgments subtest of the NADL-F short battery—assessing financial DM—and two of the six subtests within the same battery that evaluate basic numerical financial skills (see Table 3).

#### 3.6.6 Associations between DM and social cognition

Significant relationships with measures of social cognition were observed exclusively for the IGT. More advantageous performance on the IGT correlated with a greater capacity to appropriately attribute internal mental states to others, as evidenced by results from a mind-reading task (Mimura et al., 2006; Xi et al., 2015), and with improved recognition of facial emotions (Ibarretxe-Bilbao et al., 2009). Conversely, no significant correlations between measures of social cognition—specifically, Theory of Mind—and other DM tasks—i.e., the Trust Game, GDT, Everyday Moral DM task, and calculation-based social DM task—were identified (Euteneuer et al., 2009; Rosen et al., 2015; Caballero et al., 2022; Rosen et al., 2013; Xi et al., 2015; Zapf et al., 2022) (see Table 3).

#### 3.6.7 Summary

**Figure 2:**
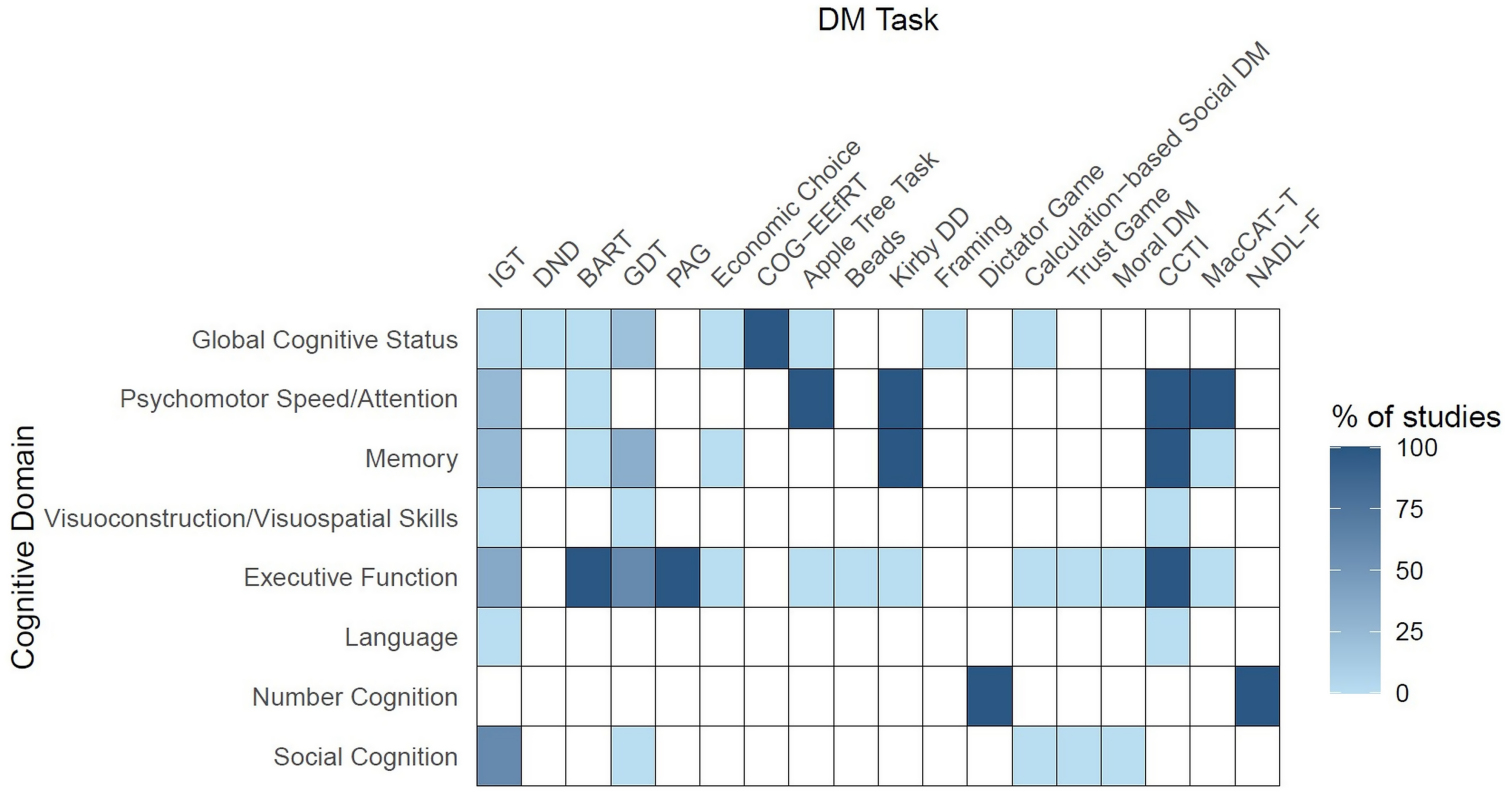
Heatmap visualizing the study availability and the proportion of studies reporting significant associations for each DM-task and cognitive domain combination in PD. Concerning study availability, white cells indicate that no studies have examined this association to date; colored cells represent combinations investigated in at least one study. The color intensity reflects the proportion of studies reporting a significant association (via correlation or regression-based analysis) relative to the total number of studies investigating the corresponding combination. Increasingly darker shades of blue indicate a higher proportion of studies reporting significant results. For more detailed information on the underlying data (i.e., which studies contribute to each cell, characteristics of specific PD samples, and the exact statistical findings of significant associations), please see Supplementary Tables S1–S5. BART, Balloon Analogue Risk Task; CCTI, Capacity to Consent to Treatment Instrument; COG-EEfRT, Cognitive adaptation of the Effort Expenditure for Rewards Task; DM, Decision-Making; DND, Deal or No Deal; GDT, Game of Dice Task; IGT, Iowa Gambling Task; Kirby DD, Kirby Delayed Discounting Questionnaire; MacCAT-T, MacArthur Competence Assessment Tool for Treatment; NADL-F, Numerical Activities of Daily Living – Financial (short battery), Financial Judgments subtest; PAG, Probability-Associated Gambling Task.

Figure 2 depicts the frequency of studies reporting significant associations between DM and cognition relative to the number of studies investigating this particular relationship. Most DM–cognition combinations are characterized by a small proportion of studies reporting significant associations (depicted in light blue). Only a few combinations (depicted in dark blue) show a majority of studies indicating at least one significant finding. In summary, inferences about relationships with multiple cognitive domains can be drawn only for a limited number of DM task (i.e., the IGT, GDT, and CCTI).

## 4 Discussion

This systematic review included 38 studies that explored the critical relationship between cognitive functioning and DM performance in individuals with PD. Deficits in DM among PD patients can have significant and wide-ranging consequences, impacting daily functioning, health-related decisions, social interactions, and overall quality of life (Gleichgerrcht et al., 2010; Caballero et al., 2022; Delazer et al., 2009). These patients often face complex choices about medical care—such as adjusting medications, considering inpatient rehabilitation, or opting for advanced therapies like DBS or pumps—but also about work and further aspects of life (Buelow et al., 2014; Dymek et al., 2001). Poor DM in these contexts can lead to adverse health outcomes, disease progression, and increased caregiver burden. Understanding the link between cognitive functions and DM performance is essential for early detection of (potential) DM impairments and for developing targeted interventions. By identifying specific cognitive deficits that may influence DM abilities, clinicians and caregivers can implement strategies to support patients, improve health outcomes, and enhance quality of life. This review synthesizes existing evidence on how various cognitive domains, such as executive functions, memory, and attention, relate to DM abilities in PD, aiming to inform clinical practice and guide future research efforts in this important area.

Several key points emerge when evaluating the findings from the perspective of DM tasks. Studies on the IGT, by far the most frequently used task, involved analyses across a broad range of outcome indices in combination with various cognitive variables. A number of associations with cognition were investigated; however, most results were non-significant. Notably, the significant findings were scattered across different cognitive domains rather than concentrated within a single area. Specifically, isolated significant associations were observed between IGT performance and global cognitive functioning, psychomotor speed, verbal memory, social cognition, and executive functions. A similar pattern was observed for the GDT, the second most commonly used DM task. Here, a small number of significant but widely dispersed results were reported, including isolated correlations with global cognitive status, verbal memory, and executive functions. The CCTI yielded several significant correlations within a single study, linking specific legal standards to attention, memory, and executive function measures. A few isolated significant associations were also observed for other DM tasks: the PAG task (with set-shifting/cognitive flexibility), the Kirby Delayed Discounting Questionnaire (with psychomotor speed and a composite memory score), the BART (with inhibition), the COG-EEfRT (with global cognitive status), the Apple Tree Task and the MacCAT-T (both with psychomotor speed), and the Dictator Game and the financial judgments subtest of the NADL-F short battery (both with numerical skills). Other DM tasks—including the Deal or No-Deal task, Economic Choice task, Beads task, Framing Paradigm, calculation-based social DM task, Trust Game, and Everyday Moral DM task—were rarely examined in relation to cognition, and none demonstrated significant associations.

Regarding the cognitive domains examined, only a limited number of studies explored the relationship between DM performance—assessed through various paradigms—and social cognition, psychomotor speed and attention, visuo-construction and visuo-spatial skills, language abilities, and numerical skills. In contrast, a relatively larger body of research investigated associations between DM tasks and global cognitive status, executive functions, and memory. Within the domain of executive functions, set-shifting/cognitive flexibility and verbal fluency emerged as the most frequently assessed subdomains, also demonstrating the highest absolute amount and proportion of significant associations with DM across all executive function measures. Conversely, other subdomains, such as conceptualization, inhibition, planning, and working memory, and composite executive function scores have been studied less extensively and yielded only sporadic significant results. Although numerous studies explored the relationship between DM tasks and global cognitive functioning, most commonly using the Mini Mental State Examination (MMSE), only three studies reported significant correlations (with the MoCA and MDRS total scores). In the memory domain, isolated significant associations were observed for general composite scores, verbal short- and long-term memory, while no significant relationships were found for visual memory. Regarding attention, numerical skills, and social cognition, the available studies and analyses were limited, with some indicating significant findings. Specifically, in the attention domain, significant associations with DM were identified for global attention measures and psychomotor speed, whereas no significant relationships emerged for sustained or divided attention. For numerical skills, only two studies examined the link with DM, both reporting some significant associations. Concerning social cognition, sporadic significant associations were observed between DM (exclusively for the IGT) and measures of emotion recognition and Theory of Mind. No significant correlations were identified between DM performance and measures of visuo-construction and visuo-spatial skills, or language abilities. Noteworthy, not all significant associations aligned with expectations. In some studies, more advantageous DM performance was linked to better cognitive functioning; in others, the opposite pattern was observed.

The results of this systematic review indicate that significant associations between DM and cognitive variables were scarce; most studies reported non-significant findings. This pattern aligns with prior reviews (Colautti et al., 2021; Poletti et al., 2011; Sinz et al., 2008; Colautti et al., 2023) and expands upon their observations through a more comprehensive approach. Most of the evidence pertained to bivariate relationships, such as correlation analyses, while fewer studies employed multivariable models (e.g., regression analyses) that account for the effects of other variables, including disease-related and/or -unrelated factors. No clear pattern emerged regarding the few significant associations identified. These significant findings were not concentrated within specific DM tasks, cognitive domains, or particular DM–cognition combinations, and should be interpreted with caution. Indeed, in nearly all cases where an association was reported as significant, at least one other study investigating the same combination found a non-significant result, indicating inconsistency across the literature. Furthermore, some results were unexpected—for example, social cognition showed a significant association with performance on the IGT but did not correlate significantly with moral DM.

An important consideration when interpreting these results—especially given the inconsistent pattern of findings—is the methodological variability among the included studies, particularly regarding the sample characteristics and the specific focus of the analyses. The PD samples examined differ in factors such as disease severity, as well as the presence of comorbidities. Furthermore, in some studies, the samples used for correlation or regression analyses comprised both patients and healthy controls. This broader inclusion complicates efforts to draw conclusions about the relationships between DM performance and cognitive functions specifically within PD patients. Additionally, many studies featured relatively small sample sizes, often sufficient to detect group-level differences (the primary focus of most investigations), but likely underpowered for identifying the smaller effect sizes typically observed in correlation analyses, which were usually secondary analyses. Moreover, these samples almost exclusively consisted of individuals with relatively well-preserved cognitive functioning, a pragmatic choice to ensure compliance with study protocols. However, this selection criterion excludes those with more pronounced deficits, thereby limiting the variability in cognitive performance and thus reducing the likelihood of detecting meaningful correlations (Zapf et al., 2022). Another point to consider is that the different outcome measures assessed across the studies for DM tasks—particularly the IGT (e.g., total net score, number of disadvantageous choices)—emphasize distinct aspects of the DM process and thus influence the likelihood of associations with specific cognitive variables (Sinz et al., 2008; Brand et al., 2007).

Several explanations have been proposed in the literature to account for the inconsistent or weak associations observed between DM and cognitive functions in PD. For instance, some authors (Colautti et al., 2023) suggest that DM and the cognitive domains assessed by routine neuropsychological tests—such as memory, executive functions, and attention—are mediated by largely distinct, only partly overlapping neural substrates. This neural dissociation may contribute to the variability in findings and the frequent absence of a strong correlation between DM performance and cognitive measures in PD patients. Furthermore, the effects of dopamine depletion in distinct brain areas, along with the influence of dopaminergic medications used in PD, introduce additional complexity. These medications often have differential effects on various cognitive functions. According to the *dopamine overdose hypothesis* (Cools et al., 2001; Vaillancourt et al., 2013), dopaminergic therapy can enhance functions primarily mediated by the dorsolateral fronto-striatal circuit—such as executive functions—by increasing dopamine levels, which are pathologically low in PD. Conversely, however, it may impair processes such as reward processing and DM, which are primarily supported by ventral striatal and orbitofrontal circuits that tend to remain relatively preserved in PD. Overstimulation of these circuits through dopaminergic medication can lead to dysfunction. Notably, other cognitive domains—such as attention and memory—appear to be largely unaffected by dopaminergic treatment. These differential effects can produce inconsistent performance patterns across cognitive domains, making it difficult to detect clear associations. In some cases, this can even result in unexpected findings, such as correlations between better cognitive functioning and poorer DM performance, or vice versa. Similarly, while DBS of the subthalamic nucleus is effective in improving motor symptoms, it can have mixed effects on cognition, enhancing certain cognitive functions but impairing others such as inhibitory control and DM (Brandt et al., 2015). The inherent fluctuations in cognition observed in PD, mostly related to variations in dopamine levels, add to this complexity. Cognitive performance, including DM, can fluctuate over time, with periods of both decline and improvement (Halhouli et al., 2022). These state-dependent fluctuations make it challenging to establish stable, consistent associations between DM and other cognitive functions. The complexity of the DM tasks themselves also contributes to these challenges. DM assessments typically involve multiple stages, processes, and factors, whereas traditional neuropsychological tests often measure isolated cognitive functions. As a result, deficits or variations in specific cognitive components may influence only particular aspects of DM rather than overall performance, thereby obscuring potential associations (Colautti et al., 2023). Finally, alterations in the cognitive processes inherently engaged during DM—such as feedback processing or stimulus–reward learning—as well as comorbid factors common in PD, including apathy, depression, anxiety, and ICDs, can significantly influence DM performance. These factors may exert a more salient influence than performance in independent cognitive domains, potentially overriding or obscuring their contribution and further complicating the relationship between DM and other cognitive functions.

A number of limitations should be acknowledged. Firstly, heterogeneity in sample characteristics (e.g., disease severity, comorbidities), cognitive measures, and DM tasks across studies poses challenges for direct comparisons and meta-analyses. However, this variability was an intentional aspect of our approach. The primary aim of this review was to capture a broad range of cognitive variables and DM scenarios, thereby providing a comprehensive overview of the potential relationships between DM abilities and cognitive functioning among individuals with PD. Unlike previous reviews (Colautti et al., 2021; Poletti et al., 2011; Sinz et al., 2008; Colautti et al., 2023), which focused on a few specific DM tasks and included only a limited number of studies, this review considered both laboratory-based gambling paradigms and more ecologically valid, real-life DM tasks, as well as the full spectrum of cognitive domains. This broader scope aimed to better reflect the variety of decision types faced by PD patients, ranging from ambiguous choices to informed medical or social decisions, and the assumption that a wide variety of cognitive functions can interact with DM. Furthermore, most original studies only conducted secondary analyses of the link between cognition and DM, often providing limited detail and pooled data that may obscure PD-specific effects. Our critical appraisal of the included studies revealed potential limitations in their methodological quality, particularly regarding small sample sizes, limited correction for multiple testing, and the use of pooled samples, which may impact the reliability and generalisability of the findings. Our review systematically distinguished between associations that pertained exclusively to PD patients and those involving combined samples, emphasizing an important aspect that has previously received limited attention in review articles. Moreover, most studies employed laboratory-based gambling paradigms, such as the IGT and the GDT, with a predominant focus on executive functions and memory. Consequently, there is limited knowledge of DM in more ecologically valid contexts and of the role of cognitive domains such as number cognition. Future research should therefore aim to address these gaps by exploring DM in real-life settings and across a broader range of cognitive functions. Another important limitation is the inconsistent assessment and reporting of medication state across studies. This variability hampers the ability to distinguish disease-related effects from medication influences. Future research should therefore focus on standardizing the assessment and reporting of medication state (e.g., comparable measures like the LEDD and ON/OFF state during the assessment), as this is essential for advancing understanding in this area. Additionally, when interpreting the results and their implications, it must be also taken into account that DM tasks represent objective instruments that may not fully capture DM in real-life, personal contexts (Dymek et al., 2001; Martin et al., 2008). This even applies to DM tasks closer to everyday life, such as the CCTI (Halhouli et al., 2022). Participants may respond differently in experimental settings than in everyday situations, where emotional factors and tangible consequences play a larger role. Although we attempted to conduct a comprehensive review, we acknowledge that studies not published in English or in German were excluded, potentially omitting relevant contributions. Furthermore, we did not perform formal assessments of publication bias, primarily due to the substantial heterogeneity among the included studies with regard to DM and cognitive measures, including task types, outcome variables, statistical analyses, and cognitive domains assessed. This diversity makes it difficult to derive a single, comparable effect size or coefficient and increases the risk of misleading conclusions. Most studies analysed the association between DM and cognition as a secondary aim, often focussing on differences between PD patients and healthy controls. Since the publication of these studies was not primarily driven by the significance of the DM-cognition relationship, evaluating potential publication bias specific to this association is challenging. Nevertheless, we acknowledge that selective reporting bias within studies, such as reporting or emphasizing certain associations or outcomes over others, remains a potential concern.

Research in neuropsychology and neuroimaging (Brand et al., 2006; Trepel et al., 2005; Schultz et al., 2008) indicates that DM under ambiguity, as examined in the initial trials of the IGT, primarily involves the *limbic loop*. This neural circuit encompasses structures such as the ventromedial prefrontal cortex, medial orbitofrontal cortex, amygdala, ventral striatum, and related neurotransmitter systems—all of which play crucial roles in emotion regulation and feedback learning. Conversely, the *cognitive loop*, comprising the dorsolateral prefrontal cortex, lateral orbitofrontal cortex, and dorsal striatum, is primarily engaged during DM under risk—such as in the later trials of the IGT or in the GDT—and supports executive functions like set shifting, inhibition, and categorization. The role of episodic memory and related medial temporal structures in DM has been controversially discussed (Brand et al., 2006). As individuals with PD may show deficits in both DM under ambiguity and DM under risk, alterations in limbic and cognitive loops have been suggested (Ryterska et al., 2013; Colautti et al., 2021; Poletti et al., 2011; Gleichgerrcht et al., 2010). Our results indicate significant correlations with executive functions and memory for both DM under ambiguity and DM under risk, while an association with social cognition was found predominantly for DM under ambiguity. While it is tempting to extrapolate from the findings of this review to deepen our understanding of the neuropsychological and neurofunctional mechanisms underlying DM processes in general and in PD in particular, the heterogeneity of the existing results and the methodological variability of the included studies currently limit such conclusions. To advance understanding in this area, larger, rigorously controlled studies are needed, particularly those on PD that integrate both experimental laboratory-based paradigms and ecologically valid DM tasks designed to mirror real-life DM scenarios. Combining comprehensive cognitive assessments across multiple domains and including more cognitively heterogeneous PD samples will help capture greater variability. This approach will facilitate a more nuanced picture of how various cognitive factors may influence DM performance, also depending on the specific DM paradigm and the processing stage under investigation. Future research should not only aim to identify potential differences in DM performance between PD patients and healthy controls but also address the link of DM with various cognitive functions as the primary outcome. Studies should explicitly consider the characteristics and manipulations of DM tasks, as these may engage distinct cognitive mechanisms. Research should also aim to clarify the impact of cognitive performance within a broader context of influencing factors, considering disease-related and -unrelated factors affecting DM in PD. Ultimately, these efforts are critical for disentangling the multifaceted factors that influence DM performance in PD, thereby informing the development of targeted interventions or support opportunities to enhance DM capacity, health outcomes, and overall quality of life in individuals with PD.

In summary, this systematic review underscores the complex and inconsistent relationship between cognitive functions and DM performance in individuals with PD. Although some studies report significant associations, most evidence is characterized by non-significant, scattered findings and considerable methodological heterogeneity. In line with prior observations (Djamshidian et al., 2012; Kobayakawa et al., 2008), these results suggest that, although DM and cognition are interconnected in PD—given the isolated significant associations—they are not entirely overlapping. Importantly, DM impairments in PD cannot be fully explained by cognitive deficits alone, indicating a partial dissociation and pointing to the influence of disease-specific factors beyond cognition. From a practical perspective, the reviewed empirical evidence (see also Pignatti et al., 2012; Poletti et al., 2009) suggests that cognitive performance should not be used as a sole proxy for DM ability in PD patients. It is important to recognize that individuals with cognitive deficits may still be capable of making adequate decisions, while cognitively preserved patients may show impaired DM performance. Given the complex interplay between DM and cognition in PD, it is crucial for patients, caregivers, and clinicians to be aware that DM impairments may occur independently of cognitive deficits and may have significant implications across various life domains, including financial, social, and health-related areas. Consequently, routine screening for DM difficulties and targeted, individualized support should be provided to address these challenges.

## Data Availability

The original contributions presented in the study are included in the article/Supplementary material, further inquiries can be directed to the corresponding author/s.
