# Supplementary material for: Cognitive correlates of decision-making in Parkinson’s disease: a systematic review
Source: Front Aging Neurosci. 2026 Jun 12;18:1840023. doi: 10.3389/fnagi.2026.1840023 (PMC13303477; doi:10.3389/fnagi.2026.1840023)
Supplement: Supplementary file 1 [file Data_Sheet_1.pdf]

Table S1. Summary of the significant (in bold) and non-significant associations between laboratory-based DM tasks under ambiguity and various cognitive measures examined in the included studies.

	Laboratory-based DM tasks under ambiguity		
Cognitive Domain	IGT	Deal or No Deal	BART
**Global cognitive status**	MMSE<br>Perretta 2005[1], Mimura 2006, Pagonabarraga 2007, Kobayakawa 2008, Euteneuer 2009, Kobayakawa 2010[3], Poletti 2010[4], Oyama 2011, Buelow 2014[5], Xi 2015, Kobayakawa 2017[2], Marques 2022[6,15], Ueda 2022<br><br>**MDRS**<br>**Pagonabarraga 2007 (total net score: ρ=−.56)**<br>Stout 2001**,** Czernecki 2002<br><br>MoCA<br>Buelow 2014[5]<br><br>DemTect<br>Euteneuer 2009<br><br>PD-CRS<br>Martínez-Horta 2013[5,15]	MoCA<br>Brandt 2015[7]	MMSE<br>Buelow 2014[5]<br><br>MoCA<br>Buelow 2014[5]
**Psycho-motor speed and attention**	Attention (MDRS)<br>Stout 2001<br><br>TMT-A<br>Sáez-Francàs 2014[8]<br><br>Stroop-1<br>Sáez-Francàs 2014[8]<br><br>**Stroop-2**<br>**Sáez-Francàs 2014[9] (total net score with number of correct responses in Stroop-2 given in 45s: r_s=.28)**<br><br>Sustained attention (PD-CRS)<br>Martínez-Horta 2013[5,15]<br><br>Sustained attention (CPT II)<br>Ibarretxe-Bilbao 2009[10]	-	TMT-A<br>Martini 2018[11]<br><br>Hayling-1<br>Martini 2018[11]<br><br>Divided attention (TAP)<br>Martini 2018[11]

**Memory**	**Digit span forward**<br>**Kobayakawa 2010[16] (number of selections from disadvantageous decks: ρ=.64)**,<br>Xi 2015, Colautti 2024<br><br>Memory (MDRS)<br>Stout 2001<br><br>Verbal memory (PD-CRS)<br>Martínez-Horta 2013[5,15]<br><br>Grober free recall<br>Czernecki 2002<br><br>**Verbal delayed free recall (RAVLT)**<br>**Pagonabarraga 2007 (total net score: ρ=−.56)**,<br>Xi 2015<br><br>Figural delayed recall (ROCFT)<br>Kobayakawa 2010[3], Kobayakawa 2017[2]	-	Composite memory score[18]<br>Martini 2018[11]
**Visuo-construction and visuo-spatial skills**	Posterior-cortical Score (PD-CRS)<br>Martínez-Horta 2013[5,15]<br><br>Hooper Visual Organization Task<br>Xi 2015<br><br>Construction (MDRS)<br>Stout 2001<br><br>Clock Copy (PD-CRS)<br>Martínez-Horta 2013[5,15]<br><br>Figure copy (ROCFT)<br>Kobayakawa 2010[3], Kobayakawa 2017[2]	-	-
**Executive functions**	**Frontal score**<br>**Czernecki 2002 (total net score: ρ=−.59)**<br><br>Fronto-subcortical score (PD-CRS)<br>Martínez-Horta 2013[5,15]<br><br>FAB<br>Poletti 2010[4], Oyama 2011, Kobayakawa 2017[2], Ueda 2022	-	Executive function (CAMCOG)<br>Martini 2018[11]<br><br>BSAT<br>Martini 2018[11]<br><br>TMT-B<br>Martini 2018[11]

	Clock drawing (PD-CRS)<br>Martínez-Horta 2013[5,15]<br><br>Maze-tracing (WISC-R)<br>Mimura 2006<br><br>Tower of London<br>Gescheidt 2012[12], Sáez-Francàs 2014[8]<br><br>Initiation/Perseveration (MDRS)<br>Stout 2001<br><br>**Semantic and/or phonemic verbal fluency**<br>**Pagonabarraga 2007 (total net score with phonemic verbal fluency: ρ=−.62 and semantic verbal fluency: ρ=−.42), Colautti 2024[17] (net score in block 1 with phonemic verbal fluency: ρ=.42),**<br>Czernecki 2002 (phon and sem), Mimura 2006 (phon and sem), Delazer 2009[13] (sem), Euteneuer 2009 (phon and sem), Xi 2015 (sem)<br><br>Action verbal fluency (PD-CRS)<br>Martínez-Horta 2013[5,15]<br><br>**Alternating verbal fluency and/or verbal fluency shifting index**<br>**Colautti 2024 (total net score with alternating verbal fluency: ρ=.31 and with verbal fluency shifting index: ρ=.35;**<br>**net score in block 4 with verbal fluency shifting index: ρ=.38),**<br>Martínez-Horta 2013[5,15] (alternating)<br><br>Conceptualization (MDRS)<br>Stout 2001<br><br>LPS-4<br>Euteneuer 2009<br><br>**(K)WCST / MCST**<br>**Ueda 2022 (total net score with number of correct categories in MCST: ρ=-.32),**<br>Czernecki 2002, Perretta 2005[1], Mimura 2006, Kobayakawa 2008, Euteneuer 2009, Kobayakawa 2010[3], Oyama 2011, Kobayakawa 2017		**Go-NoGo (TAP)**<br>**Martini 2018[11] (discrepancy score with Go-NoGo false alarms, r_s=−.34)**<br><br>Hayling-2<br>Martini 2018[11]

	**TMT-B(-A)**<br>**Delazer 2009[14] (overall frequency of shifts between advantageous and disadvantageous decks with response times in TMT-B: r=.62),**<br>Sáez-Francàs 2014[8], Ueda 2022<br><br>OMO<br>Delazer 2009[13]<br><br>Go-NoGo (FAB)<br>Delazer 2009[13]<br><br>**Stroop**<br>**Colautti 2024 (net score in block 1 with Stroop test errors: ρ=.39),**<br>Perretta 2005[1], Mimura 2006, Pagonabarraga 2007, Gescheidt 2012[12], Sáez-Francàs 2014[8], Xi 2015, Ueda 2022,<br><br>Working memory (PD-CRS)<br>Martínez-Horta 2013[5,15]<br><br>**Digit span backward**<br>**Ibarretxe-Bilbao 2009[10] (total net score: r=.48), Colautti 2024 (total net score: ρ=-.39, net score in block 2: ρ=−.39, net score in block 3: ρ=−.33, net score in block 4: ρ=− .35, net score in block 5: ρ=−.37),**<br>Delazer 2009[13], Pagonabarraga 2007, Euteneuer 2009, Kobayakawa 2010[3], Xi 2015		
**Language**	Confrontation naming (PD-CRS)<br>Martínez-Horta 2013[5,15]	-	-
**Numerical skills**	-	-	-
**Social Cognition**	Facial Emotion Recognition Test<br>Ueda 2022<br><br>**Ekman test**<br>**Ibarretxe-Bilbao 2009[10] (total net score with Ekman total score: r=.41)**<br><br>**RMET**<br>**Mimura 2006 (amount left with RMET mental-state condition: r_s=.59), Xi 2015 (total number of advantageous selections with emotion recognition / mind reading in RMET: r=.58),**<br>Euteneuer 2009	-	-

Notes: Only the evidence from the original studies including analyses with Parkinson's disease (PD) patients is presented in this table. Analyses that include specific PD subgroups or pooled samples are indicated with footnotes; if no such specification is provided, the results refer to a PD sample without systematic presence of comorbidities. Only the decision-making (DM) and cognitive tasks assessed in the original studies that met our inclusion criteria and that were included in association analyses (i.e., correlation or regression-based analyses) are listed here. The grouping of DM tasks emphasizes the primary focus and context of each task rather than strict or mutually exclusive categories. Entries highlighted in bold indicate a significant association. It is important to acknowledge that, across studies, different outcome variables were employed for certain tasks – particularly the Iowa Gambling Task (IGT) and some executive function tasks – which can influence the results in terms of significance and the direction of associations (positive vs. negative). To enhance clarity, the table presents the tasks incorporated into statistical analyses; details about the specific outcome variables assessed are provided only in cases of significant associations (this applies to the DM task, as these were frequently evaluated using different outcome variables; for the cognitive tasks, detailed information on outcome measures was reported only when a test allows multiple modes of scoring, which can influence the direction of associations – for example, the number of errors versus the number of correct responses within a limited time in the Stroop task). Unless otherwise specified, the results refer to bivariate relationships (i.e., correlation analyses). Some studies reported multiple analyses for a given association (e.g., different outcome variables within cognitive or DM tasks, or using alternative statistical models). In rare cases, findings for a given association were both significant and non-significant across different outcome variables or analyses. In such instances, we reported the significant result, as it suggests evidence of an association – even if not consistently observed across all measures. An exception is Buelow et al. (2014), which reported only one significant correlation for a minor sub-variable of the IGT among many non-significant results; this was summarized as non-significant, as the isolated sub-finding was unlikely to indicate a reliable association.

[1] The results are not significant for both early and later PD patients separately.

[2] It is not specified (and it could not be inferred) whether the analysis was carried out for the PD group separately and/or for the total sample (usually PD + HC).

[3] The results are not significant for both the original and modified version of the IGT.

[4] The analysis was carried out for de-novo, untreated PD patients.

[5] The analysis was carried out for PD patients with apathy and PD patients without apathy together.

[6] The analysis was carried out both for the whole sample (PD with RBD, PD without RBD, and HC together) as well as for PD patients exclusively (PD with RBD and PD without RBD together).

[7] The analysis was carried out for the whole sample (PD with DBS, PD without DBS, and HC together).

[8] The results are not significant for both fatigued and non-fatigued PD patients separately and combined.

[9] The results are significant for non-fatigued PD patients but not for fatigued PD patients.

[10] The analysis was carried out for PD patients and HC together.

[11] The analysis was carried out for the whole sample (PD with ICD, PD without ICD, and HC together).

[12] The analysis was carried out for early-onset PD patients.

[13] The results are not significant for both PD and PDD patients separately.

[14] The results are significant for PD patients but not PDD patients.

[15] The associations were evaluated within a multivariable model (e.g. multiple regression analysis, GEE). Thus, they do not represent the isolated bivariate associations, but rather the contribution of the cognitive variable in presence of or after adjusting for additional covariates (e.g., testing whether the cognitive variable adds incremental predictive value beyond other predictors).

[16] The results are significant for the modified version of the IGT but not for the original version.

[17] The results are significant for phonemic verbal fluency, but not for semantic verbal fluency.

[18] Composite memory score = Logical memory immediate and delayed recall WMS (Wechsler Memory Scale) + Memory subtest CAMCOG.

Abbreviations: BART = Balloon Analogue Risk Task; BSAT = Brixton Spatial Anticipation Test; CAMCOG = Cambridge Cognition Examination; CPT II = Conners' Continuous Performance Test II; DBS = Deep Brain Stimulation; DemTect = Demenz-Detektion; DM = Decision-Making; FAB = Frontal Assessment Battery; HC = Healthy Controls; ICD = Impulse control disorder; IGT = Iowa Gambling Task; (K)WCST = (Keio) Wisconsin Card Sorting Test; LPS = Leistungsprüfsystem; MCST = Modified Card Sorting Test; MDRS = Mattis Dementia Rating Scale; MMSE = Mini Mental State Examination; MoCA = Montreal Cognitive Assessment; OMO = Odd Man Out; PD = Parkinson's Disease; PD-CRS = Parkinson's Disease-Cognitive Rating Scale; PDD = Parkinson's Disease Dementia; RAVLT = Rey Auditory Verbal Learning Test; RBD = REM (Rapid eye movement) sleep behavior disorder; RMET = Reading the Mind in the Eyes Test; ROCFT = Rey-Osterrieth Complex Figure Test; TMT = Trail Making Test; TAP = Testbatterie zur Aufmerksamkeitsprüfung; WISC-R = Wechsler Intelligence Scale for Children – revised.

Table S2. Summary of the significant (in bold) and non-significant associations between laboratory-based DM tasks under risk and various cognitive measures examined in the included studies.

	Laboratory-based DM tasks under risk		
Cognitive Domain	GDT	PAG	Economic Choice
**Global cognitive status**	MMSE<br>Brand 2004, Euteneuer 2009, Boller 2014[1], Xi 2015<br><br>**MoCA**<br>**Brandt 2015[2] (number of 1-side bets / riskiest option: ρ=−.45; final capital: ρ=.33)**<br><br>MDRS<br>Boller 2014[1]<br><br>DemTect<br>Brand 2004, Euteneuer 2009<br><br>PANDA<br>Boller 2014[1]	-	MMSE<br>Kobayashi 2019[3,7]
**Psycho-motor speed and attention**	-	-	-
**Memory**	Digit span forward<br>Xi 2015, Colautti 2024<br><br>**Verbal delayed free recall (RAVLT)**<br>**Xi 2015 (final capital: r=.57)**<br><br>Word list (DemTect)<br>Brand 2004	-	Figural delayed recall (ROCFT)<br>Kobayashi 2019[3,7]
**Visuo-construction and visuo-spatial skills**	Hooper Visual Organization Task<br>Xi 2015<br><br>LPS-7<br>Brand 2004	-	-
**Executive functions**	**Semantic and/or phonemic verbal fluency**<br>**Euteneuer 2009[4] (final capital with phon: r=.48),**<br>Brand 2004 (phon and sem), Boller 2014[1] (phon), Xi 2015 (sem), Colautti 2024 (phon and sem)	Semantic verbal fluency<br>Delazer 2009[5]<br><br>TMT-B<br>Delazer 2009[5]	FAB<br>Kobayashi 2019[3,7]

	Alternating verbal fluency and verbal fluency shifting index<br>Colautti 2024<br><br>LPS-4<br>Brand 2004, Euteneuer 2009, Boller 2014[1]<br><br>**MCST**<br>**Brand 2004 (frequency of disadvantageous decisions with MCST non-perseverative errors raw scores: r=.51 or t-scores: r=−.46), Euteneuer 2009 (net score with MCST correct numbers: r=.51 and MCST perseverative errors: r=-.50; final capital with MCST correct numbers: r=.56 and MCST perseverative errors: r=−.45 and MCST non-perseverative errors r=-.48)**<br><br>**Stroop**<br>**Colautti 2024 (net score with Stroop time: ρ=-.37; riskiest option with Stroop time: ρ=.39),**<br>Xi 2015<br><br>Digit span backward<br>Brand 2004, Euteneuer 2009, Xi 2015, Colautti 2024	**OMO**<br>**Delazer 2009[6] (frequency of gambles in the low probability condition with errors in OMO: r=.68)**<br><br>Go-NoGo (FAB)<br>Delazer 2009[5]<br><br>Digit span backward<br>Delazer 2009[5]	
**Language**	-	-	-
**Numerical skills**	-	-	-
**Social Cognition**	RMET<br>Euteneuer 2009, Xi 2015	-	-

Notes: Only the evidence from the original studies including analyses with Parkinson's disease (PD) patients is presented in this table. Analyses that include specific PD subgroups or pooled samples are indicated with footnotes; if no such specification is provided, the results refer to a PD sample without systematic presence of comorbidities. Only the decision-making (DM) and cognitive tasks assessed in the original studies that met our inclusion criteria and that were included in association analyses (i.e., correlation or regression-based analyses) are listed here. The grouping of DM tasks emphasizes the primary focus and context of each task rather than strict or mutually exclusive categories. Entries highlighted in bold indicate a significant association. It is important to acknowledge that, across studies, different outcome variables were employed for certain tasks, which can influence the results in terms of significance and the direction of associations (positive vs. negative). To enhance clarity, the table presents the tasks incorporated into statistical analyses; details about the specific outcome variables assessed are given only in cases of significant associations (this applies to the DM task, as these were frequently evaluated using different outcome variables; for the cognitive tasks, detailed information on outcome measures was reported only when a test allows multiple modes of scoring, which can influence the direction of associations – for example, the number of errors versus the number of correct responses within a limited time in the Stroop task). Unless otherwise specified, the results refer to bivariate relationships (i.e., correlation analyses). Some studies reported multiple analyses for a given association (e.g., different outcome variables within cognitive or DM tasks, or using alternative statistical models). In rare cases, findings for a given association were both significant and non-significant across different outcome variables or analyses. In such instances, we reported the significant result, as it suggests evidence of an association – even if not consistently observed across all measures.

[1] The analysis was carried out for PD patients with DBS.

[2] The analysis was carried out for the whole sample (PD with DBS, PD without DBS, and HC together).

[3] The analysis was carried out for PD patients with ICD and PD patients without ICD together.
[4] The results are significant for phonemic verbal fluency, but not for semantic verbal fluency.
[5] The results are not significant for both PD and PDD patients separately.
[6] The results are significant for PD patients but not PDD patients.
[7] The associations were evaluated within a multivariable model (e.g. multiple regression analysis). Thus, they do not represent the isolated bivariate associations, but rather the contribution of the cognitive variable in presence of or after adjusting for additional covariates (e.g., testing whether the cognitive variable adds incremental predictive value beyond other predictors).

Abbreviations: DBS = Deep Brain Stimulation; DemTect = Demenz-Detektion; DM = Decision-Making; FAB = Frontal Assessment Battery; GDT = Game of Dice Task; HC = Healthy Controls; ICD = Impulse control disorder; LPS = Leistungsprüfsystem; MCST = Modified Card Sorting Test; MDRS = Mattis Dementia Rating Scale; MMSE = Mini Mental State Examination; MoCA = Montreal Cognitive Assessment; OMO = Odd Man Out; PAG = Probability-Associated Gambling Task; PANDA = Parkinson Neuropsychometric Dementia Assessment; PD = Parkinson’s Disease; PDD = Parkinson’s Disease Dementia; RAVLT = Rey Auditory Verbal Learning Test; RMET = Reading the Mind in the Eyes Test; ROCFT = Rey-Osterrieth Complex Figure Test; TMT = Trail Making Test.

Table S3. Summary of the significant (in bold) and non-significant associations between different laboratory-based DM tasks and various cognitive measures examined in the included studies.

	Other laboratory-based DM tasks				
Cognitive Domain	COG-EEfRT	Apple Tree Task	Beads	Kirby Delayed Discounting	Framing Paradigm
**Global cognitive status**	**MoCA**<br>**Scott 2025[1] (frequency of hard task selections in high probability trials: r=.26)**	MoCA<br>Amstutz 2025[2,8]	-	-	MoCA<br>Brandt 2015[3]
**Psycho-motor speed and attention**	-	TMT-A<br>Amstutz 2025[2,8]<br><br>**Symbol Search (WAIS-IV)**<br>**Amstutz 2025[2,8] (Symbol Search z-score as a significant predictor for reaction times in DM: Estimate = -.28)**	-	TMT-A<br>Martini 2018[4]<br><br>**Hayling-1**<br>**Martini 2018[4] (total score with Hayling Section 1 scaled score: r=.33)**<br><br>Divided attention (TAP)<br>Martini 2018[4]	-
**Memory**	-	-	-	**Composite memory score[9]**<br>**Martini 2018[4] (total score: r=.41)**	-
**Visuo-construc-tion and visuo-spatial skills**	-	-	-	-	-
**Executive functions**	-	Stroop<br>Amstutz 2025[2,8]	Go-NoGo (FAB)<br>de Rezende Costa 2016[5,6]<br><br>Working memory task<br>Djamshidian 2012[7]	Executive function (CAMCOG)<br>Martini 2018[4]<br><br>BSAT<br>Martini 2018[4]<br><br>TMT-B<br>Martini 2018[4]<br><br>Go-NoGo (TAP)<br>Martini 2018[4]<br><br>Hayling-2<br>Martini 2018[4]	-
**Language**	-	-	-	-	-

**Numerical skills**	-	-	-	-	-
**Social Cognition**	-	-	-	-	-

Notes: Only the evidence from the original studies including analyses with Parkinson's disease (PD) patients is presented in this table. Analyses that include specific PD subgroups or pooled samples are indicated with footnotes; if no such specification is provided, the results refer to a PD sample without systematic presence of comorbidities. Only the decision-making (DM) and cognitive tasks assessed in the original studies that met our inclusion criteria and were included in association analyses (i.e., correlation or regression-based analyses) are listed here. The grouping of DM tasks emphasizes the primary focus and context of each task rather than strict or mutually exclusive categories. To enhance clarity, the table presents the tasks incorporated into statistical analyses; details about the specific outcome variables assessed are given only in cases of significant associations (this applies to the DM task, as these were frequently evaluated using different outcome variables; for the cognitive tasks, detailed information on outcome measures was reported only when a test allows multiple modes of scoring, which can influence the direction of associations – for example, the number of errors versus the number of correct responses within a limited time in the Stroop task). Entries highlighted in bold indicate a significant association. Unless otherwise specified, the results refer to bivariate relationships (i.e., correlation analyses). Some studies reported multiple analyses for a given association (e.g., different outcome variables within cognitive or DM tasks, or using alternative statistical models). In rare cases, findings for a given association were both significant and non-significant across different outcome variables or analyses. In such instances, we reported the significant result, as it suggests evidence of an association – even if not consistently observed across all measures.

[1] The analysis was carried out for the whole PD sample (PD with and without motivational disturbances (i.e. apathy and/or ICD)).
[2] The analysis was carried out on a combined sample of PD patients with recent (including pre- and postoperative data) and with chronic DBS, with approximately half of both subgroups with additional ICD.
[3] The analysis was carried out for the whole sample (PD with DBS, PD without DBS, and HC together).
[4] The analysis was carried out for the whole sample (PD with ICD, PD without ICD, and HC together).
[5] It is not specified (and it could not be inferred) whether the analysis was carried out for the PD group separately and/or for the total sample (usually PD + HC).
[6] The analysis was carried out for de-novo, untreated PD patients.
[7] The results are not significant for both PD with ICB and PD without ICB separately.
[8] The associations were evaluated within a multivariable model (e.g. linear mixed-effects model). Thus, they do not represent the isolated bivariate associations, but rather the contribution of the cognitive variable in presence of or after adjusting for additional covariates (e.g., testing whether the cognitive variable adds incremental predictive value beyond other predictors).
[9] Composite memory score = Logical memory immediate and delayed recall WMS (Wechsler Memory Scale) + Memory subtest CAMCOG.

Abbreviations: BSAT = Brixton Spatial Anticipation Test; CAMCOG = Cambridge Cognition Examination; COG-EEfRT = cognitive adaptation of the Effort Expenditure for Rewards Task; DBS = Deep Brain Stimulation; DM = Decision-Making; FAB = Frontal Assessment Battery; HC = Healthy Controls; ICB = Impulsive-compulsive behavior; ICD = Impulse control disorder; MoCA = Montreal Cognitive Assessment; PD = Parkinson's Disease; TMT = Trail Making Test; TAP = Testbatterie zur Aufmerksamkeitsprüfung; WAIS-IV = Wechsler Adult Intelligence Scale - Fourth Edition.

Table S4. Summary of the significant (in bold) and non-significant associations between social DM tasks and various cognitive measures examined in the included studies.

	Social DM tasks			
Cognitive Domain	Dictator Game	Calculation-based Social DM	Trust Game	Everyday Moral DM
**Global cognitive status**	-	PANDA<br>Zapf 2022	-	-
**Psychomotor speed and attention**	-	-	-	-
**Memory**	-	-	-	-
**Visuo-construction and visuo-spatial skills**	-	-	-	-
**Executive functions**	-	MCST<br>Zapf 2022<br><br>TMT-B/A<br>Zapf 2022	Semantic verbal fluency<br>Caballero 2022	Key Search Test (BADS)<br>Rosen 2015<br><br>Semantic and phonemic verbal fluency<br>Rosen 2013<br><br>LPS-4<br>Rosen 2015<br><br>MCST<br>Rosen 2013, Rosen 2015<br><br>TMT- B-A<br>Rosen 2015
**Language**	-	-	-	-
**Numerical skills**	**Number pair bisection task**<br>**Arshad 2017 (mean monetary value donated with number pair bisection error/bias: R^2=.83)**	-	-	-
**Social Cognition**	-	RMET<br>Zapf 2022	Faux-Pas Test<br>Caballero 2022	RMET<br>Rosen 2013, Rosen 2015

Notes: Only the evidence from the original studies including analyses with Parkinson's disease (PD) patients is presented in this table. Analyses that include specific PD subgroups or pooled samples are indicated with footnotes; if no such specification is provided, the results refer to a PD sample without systematic presence of comorbidities. Only the decision-making (DM) and cognitive tasks assessed in the original studies that met our inclusion criteria and were included in association analyses (i.e., correlation or regression-based

analyses) are listed here. The grouping of DM tasks emphasizes the primary focus and context of each task rather than strict or mutually exclusive categories. To enhance clarity, the table presents the tasks incorporated into statistical analyses; details about the specific outcome variables assessed are given only in cases of significant associations. Entries highlighted in bold indicate a significant association. Unless otherwise specified, the results refer to bivariate relationships (i.e., correlation analyses). Some studies reported multiple analyses for a given association (e.g., different outcome variables within cognitive or DM tasks, or using alternative statistical models). In rare cases, findings for a given association were both significant and non-significant across different outcome variables or analyses. In such instances, we reported the significant result, as it suggests evidence of an association – even if not consistently observed across all measures.

Abbreviations: BADS = Behavioural Assessment of the Dysexecutive Syndrome; DM = Decision-Making; LPS = Leistungsprüfsystem; MCST = Modified Card Sorting Test; PANDA = Parkinson Neuropsychometric Dementia Assessment; RMET = Reading the Mind in the Eyes Test; TMT = Trail Making Test.

Table S5. Summary of the significant (in bold) and non-significant associations between applied/domain-specific DM tasks and various cognitive measures examined in the included studies.

	Applied / Domain-specific DM tasks		
	Medical DM (capacity)		Financial DM
Cognitive Domain	CCTI	MacCAT-T	NADL-F: Financial Judgments
**Global cognitive status**	-	-	-
**Psychomotor speed and attention**	**Attention (DRS)**<br>**Dymek 2001[1] (S1: r=.58; S4: r=.58)**<br><br>Mental Control (WMS-R)<br>Dymek 2001[1]<br><br>Digit span forward + backward total (WMS-R)<br>Dymek 2001[1]<br><br>TMT-A<br>Dymek 2001[1]	**Digit Trial (CST)**<br>**Eygelshoven 2017[2] (significant predictor for MacCAT-T total score across all 4 subscales: R^2=.13)**<br><br>Stroop - Word card<br>Eygelshoven 2017[2]<br><br>Letter-Digit Substitution Test<br>Eygelshoven 2017[2]	-
**Memory**	**Memory (DRS)**<br>**Dymek 2001[1] (S1: r=.73; S5: r=.71)**<br><br>Logical memory I (WMS-R)<br>Dymek 2001[1]<br><br>**Logical memory II (WMS-R)**<br>**Dymek 2001[1] (S4: r=.48; S5: r=.65)**	Rivermead Behavioural Memory Test<br>Eygelshoven 2017[2]<br><br>Digit span forward<br>Eygelshoven 2017[2]<br><br>Verbal Learning Test<br>Eygelshoven 2017[2]	-
**Visuo-construction and visuo-spatial skills**	Construction (DRS)<br>Dymek 2001[1]	-	-
**Executive functions**	**EXIT-25**<br>**Dymek 2001[1] (S1: r=−.53; S4: r=−.67; S5: r=−.75)**<br><br>Initiation/Perseveration (DRS)<br>Dymek 2001[1]<br><br>Semantic and phonemic verbal fluency<br>Dymek 2001[1]	Phonemic verbal fluency<br>Eygelshoven 2017[2]<br><br>Shift and Digit trials (CST) divided<br>Eygelshoven 2017[2]<br><br>Stroop	-

	**Comprehension (WAIS-R)**<br>**Dymek 2001[1] (S1: r=.67, S5: r=.70)**<br><br>Similarities (WAIS-R)<br>Dymek 2001[1]<br><br>Conceptualization (DRS)<br>Dymek 2001[1]<br><br>**TMT-B**<br>**Dymek 2001[1] (S4: r=−.60)**	Eygelshoven 2017[2]<br><br>Digit span backward<br>Eygelshoven 2017[2]	
**Language**	Boston Naming Test<br>Dymek 2001[1]<br><br>Simple auditory comprehension test<br>Dymek 2001[1]<br><br>Token test<br>Dymek 2001[1]	-	-
**Numerical skills**	-	-	**NADL-F subtests:**<br>**Counting currencies**<br>**Danesin 2022 (Financial Judgments subtest score: β=.40, partial r=.32)**<br>**Item purchase**<br>**Danesin 2022 (Financial Judgments subtest score: β=−.35, partial r=−.29)**<br>Reading abilities<br>Danesin 2022[3]<br>Bill payments<br>Danesin 2022[3]<br>Percentages<br>Danesin 2022[3]<br>Financial concepts<br>Danesin 2022[3]
**Social Cognition**	-	-	-

Notes: Only the evidence from the original studies including analyses with Parkinson's disease (PD) patients is presented in this table. Analyses that include specific PD subgroups or pooled samples are indicated with footnotes; if no such specification is provided, the results refer to a PD sample without systematic presence of comorbidities. Only the decision-making (DM) and cognitive tasks assessed in the original studies that met our inclusion criteria and were included in association analyses (i.e., correlation or regression-based analyses) are listed here. The grouping of DM tasks emphasizes the primary focus and context of each task rather than strict or mutually exclusive categories. To enhance clarity, the table presents the tasks incorporated into statistical analyses; details about the specific outcome variables assessed are given only in cases of significant associations. Entries highlighted in bold indicate a significant association. Unless otherwise specified, the results refer to bivariate relationships (i.e., correlation analyses). Some studies reported multiple

analyses for a given association (e.g., different outcome variables within cognitive or DM tasks, or using alternative statistical models). In rare cases, findings for a given association were both significant and non-significant across different outcome variables or analyses. In such instances, we reported the significant result, as it suggests evidence of an association – even if not consistently observed across all measures.

[1] The analysis was carried out for PD patients with (overall mild) cognitive impairment. The CCTI standards are defined as follows: S1 = evidencing choice; S2 = reasonable choice; S3 = appreciate consequences; S4 = rational reasons; S5 = understand treatment. Importantly, in the original study (Dymek et al. 2001), for each CCTI standard, only the four strongest correlations with cognitive variables were reported. As such, it remains unclear whether the remaining cognitive variables exhibited non-significant correlations or whether significant but weaker correlations were found. Therefore, in the context of the results of this study, it cannot be ruled out that cognitive tasks currently marked as non-significant (not bolded) may, in fact, have yielded significant associations. Notably, all of the non-significant cognitive measures (not bolded) pertain to associations with the CCTI standards S2 and S3 (as these two standards showed no significant correlations with any cognitive variables). However, it cannot be excluded that standards S1, S4, and S5 exhibited significant (though weak) correlations with these cognitive variables.

[2] The results stem from a multiple linear regression analysis: the CST Digit Trial emerged as a significant predictor; after accounting for this, all other cognitive variables were non-significant predictors (stepwise regression).

[3] Results are not significant either in the multivariable model (multiple regression) or in the bivariate analysis (χ^2 test; co-occurrence of financial DM deficits and financial/numerical abilities deficits).

Abbreviations: CCTI = Capacity to Consent to Treatment Instrument; CST = Concept Shifting Test; DM = Decision-Making; DRS = Dementia Rating Scale; EXIT-25 = Executive Interview; MacCAT-T = MacArthur Competence Assessment Tool for Treatment; NADL-F = Numerical Activities of Daily Living – Financial (short battery); PD = Parkinson's Disease; TMT = Trail Making Test; WAIS-R = Wechsler Adult Intelligence Scale revised; WMS-R = Wechsler Memory Scale revised.

Table S6. Number of studies investigating a specific DM-cognition combination.

	Laboratory-based DM tasks											Social DM				Applied / Domain-specific DM		
	DM under ambiguity			DM under risk			Others									MDM(C)		FDM
Cognitive Domain	IGT (n=21)	Deal or No Deal (n=1)	BART (n=2)	GDT (n=6)	PAG (n=1)	Eco-nomic Choice (n=1)	COG-EEfRT (n=1)	Apple Tree Task (n=1)	Beads (n=2)	Kirby DD (n=1)	Framing Paradigm (n=1)	Dictator Game (n=1)	Calculation-based social DM (n=1)	Trust Game (n=1)	EMDM (n=2)	CCTI (n=1)	Mac CAT-T (n=1)	NADL-F: FJ (n=1)
Global cognitive status (n=23)	16	1	1	5	-	1	1	1	-	-	1	-	1	-	-	-	-	-
Psycho-motor speed / attention (n=8)	4	-	1	-	-	-	-	1	-	1	-	-	-	-	-	1	1	-
Memory (n=13)	8	-	1	3	-	1	-	-	-	1	-	-	-	-	-	1	1	-
Visuo-construction and -spatial skills (n=7)	5	-	-	2	-	-	-	-	-	-	-	-	-	-	-	1	-	-
Executive functions (n=32)	19	-	1	5	1	1	-	1	2	1	-	-	1	1	2	1	1	-
Language (n=2)	1	-	-	-	-	-	-	-	-	-	-	-	-	-	-	1	-	-
Numerical skills (n=2)	-	-	-	-	-	-	-	-	-	-	-	1	-	-	-	-	-	1
Social cognition (n=9)	5	-	-	2	-	-	-	-	-	-	-	-	1	1	2	-	-	-

Abbreviations: BART = Balloon Analogue Risk Task; CCTI = Capacity to Consent to Treatment Instrument; COG-EEfRT = cognitive adaptation of the Effort Expenditure for Rewards Task; DM = Decision-Making; EMDM = Everyday Moral Decision-Making Task; FDM = Financial Decision-Making; GDT = Game of Dice Task; IGT = Iowa Gambling Task; Kirby DD = Kirby Delayed Discounting Questionnaire; MacCAT-T = MacArthur Competence Assessment Tool for Treatment; MDM(C) = Medical Decision-Making (capacity); n = Number; NADL-F: FJ = Numerical Activities of Daily Living – Financial (short battery), Financial Judgments subtest; PAG = Probability-Associated Gambling Task.

Figure S1. Number of studies using a specific decision-making task (panel A) and investigating a specific cognitive domain (panel B).

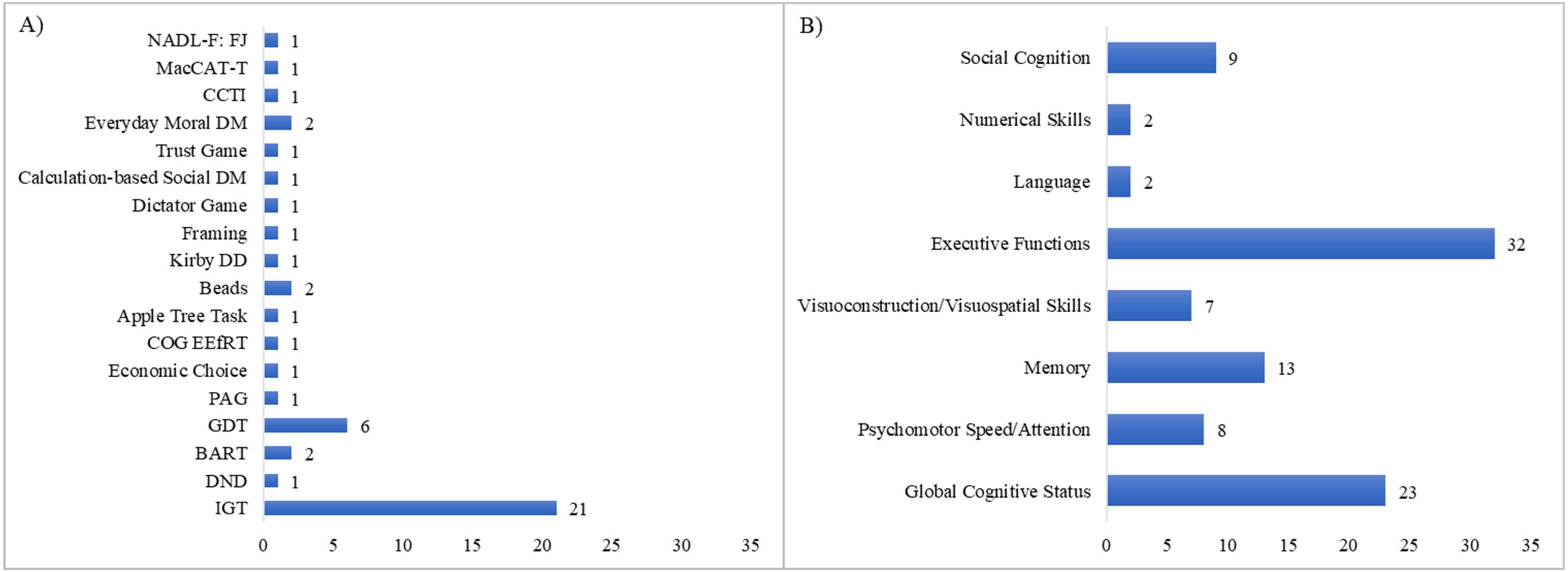

Abbreviations: BART = Balloon Analogue Risk Task; CCTI = Capacity to Consent to Treatment Instrument; COG EEfRT = Cognitive Effort Expenditure for Rewards Task; DD = Delayed Discounting; DM = Decision-Making; DND = Deal or no Deal; GDT = Game of Dice Task; IGT = Iowa Gambling Task; MacCAT-T = MacArthur Competence Assessment Tool for Treatment; NADL-F: FJ = Numerical Activities of Daily Living – Financial: Financial Judgement subtest (short battery); PAG = Probability-Associated Gambling Task.

Table S7. Critical appraisal and risk of bias assessment of included studies using the JBI critical appraisal checklist for analytical cross-sectional studies.

First author	Publication year	Q1	Q2	Q3	Q4	Q5	Q6	Q7	Q8
Amstutz	2025	● green	● yellow	● green	● green	● green	● green	● yellow	● green
Arshad	2017	● yellow	● green	● green	● green	● green	● green	● yellow	● yellow
Boller	2014	● green	● green	● green	● green	● green	● yellow	● yellow	● green
Brand	2004	● green	● yellow	● green	● green	● green	● green	● yellow	● yellow
Brandt	2015	● green	● green	● green	● green	● green	● green	● yellow	● yellow
Buelow	2014	● green	● green	● green	● green	● green	● green	● yellow	● yellow
Caballero	2022	● yellow	● red	● green	● green	● green	● yellow	● green	● yellow
Colautti	2024	● green	● green	● green	● green	● green	● green	● yellow	● green
Czernecki	2002	● green	● green	● green	● green	● green	● green	● yellow	● green
Danesin	2022	● green	● green	● green	● green	● yellow	● green	● green	● yellow
Delazer	2009	● yellow	● green	● green	● green	● green	● yellow	● green	● green
de Rezende Costa	2016	● green	● green	● green	● green	● green	● yellow	● yellow	● green

Djamshidian	2012	yellow	green	red	green	green	green	yellow	green
Dymek	2001	green	yellow	green	green	green	yellow	yellow	yellow
Euteneuer	2009	green	yellow	green	green	green	yellow	yellow	yellow
Eygelshoven	2017	green	green	green	green	green	green	yellow	green
Gescheidt	2012	green	green	green	green	green	yellow	yellow	green
Ibarretxe-Bilbao	2009	green	green	green	green	green	green	yellow	yellow
Kobayakawa	2008	yellow	red	green	green	green	yellow	yellow	green
Kobayakawa	2010	red	red	green	green	yellow	yellow	yellow	green
Kobayakawa	2017	red	red	green	green	green	green	yellow	yellow
Kobayashi	2019	green	green	green	green	green	green	yellow	green
Marques	2022	green	green	green	green	green	green	yellow	yellow
Martínez-Horta	2013	green	green	green	green	green	green	yellow	green
Martini	2018	green	green	green	green	green	yellow	yellow	green
Mimura	2006	red	yellow	yellow	green	yellow	yellow	yellow	yellow

Oyama	2011	🟡	🟢	🟢	🟢	🟢	🟡	🟡	🟢
Pagonabarraga	2007	🟢	🟢	🟢	🟢	🟢	🟡	🟡	🟢
Perretta	2005	🟢	🟡	🟢	🟢	🟢	🟢	🟡	🟢
Poletti	2010	🟡	🟢	🟢	🟢	🟢	🟡	🟡	🟡
Rosen	2013	🟢	🟡	🟢	🟢	🟢	🟢	🟢	🟢
Rosen	2015	🟢	🟢	🟢	🟢	🟢	🟢	🟢	🟢
Sáez-Francàs	2014	🟡	🔴	🟢	🟢	🟢	🟢	🟡	🟢
Scott	2025	🟢	🟢	🟢	🟢	🟢	🟢	🟡	🟡
Stout	2001	🟡	🔴	🟢	🟢	🟢	🟢	🟡	🟡
Ueda	2022	🟢	🟢	🟢	🟢	🟢	🟢	🟡	🟢
Xi	2015	🟡	🟡	🟢	🟢	🟢	🟢	🟡	🟡
Zapf	2022	🟢	🟢	🟢	🟢	🟢	🟢	🟡	🟢

Notes: The critical appraisal and risk of bias assessment were conducted using the JBI critical appraisal checklist for analytical cross-sectional studies. Q1 – Q8 correspond to the JBI questions (see below), related to specific categories of validity and domains of bias. Green circles indicate a “yes” rating (criterion fulfilled / low risk of bias), red circles indicate a “no” rating (criterion not fulfilled / high risk of bias), and yellow circles indicate an “unclear” rating (criterion partly fulfilled or met with some methodological concerns / unclear or insufficient reporting / moderate or unclear risk of bias).

Q1 = Were the criteria for inclusion in the sample clearly defined?
Q2 = Were objective, standard criteria used for measurement of the condition? *(in the context of this review, the term "condition" refers to the Parkinson's Disease diagnosis)*
Q3 = Was the exposure measured in a valid and reliable way? *(in the context of this review, the term "exposure" was operationalized as cognition)*
Q4 = Were the outcomes measured in a valid and reliable way? *(in the context of this review, the term "outcomes" was operationalized as decision-making)*
Q5 = Were confounding factors identified?
Q6 = Were strategies to deal with confounding factors stated?
Q7 = Was appropriate statistical analysis used? *(in the context of this review, the appraisal focused on statistical analyses investigating the association between cognition and decision-making, with attention not only to whether the appropriate statistical analyses had been applied but also to the robustness and methodological rigor of these analyses)*
Q8 = Were the study subjects and the setting described in detail?

Figure S2. Summary of the critical appraisal and risk of bias assessment using the JBI critical appraisal checklist for analytical cross-sectional studies.

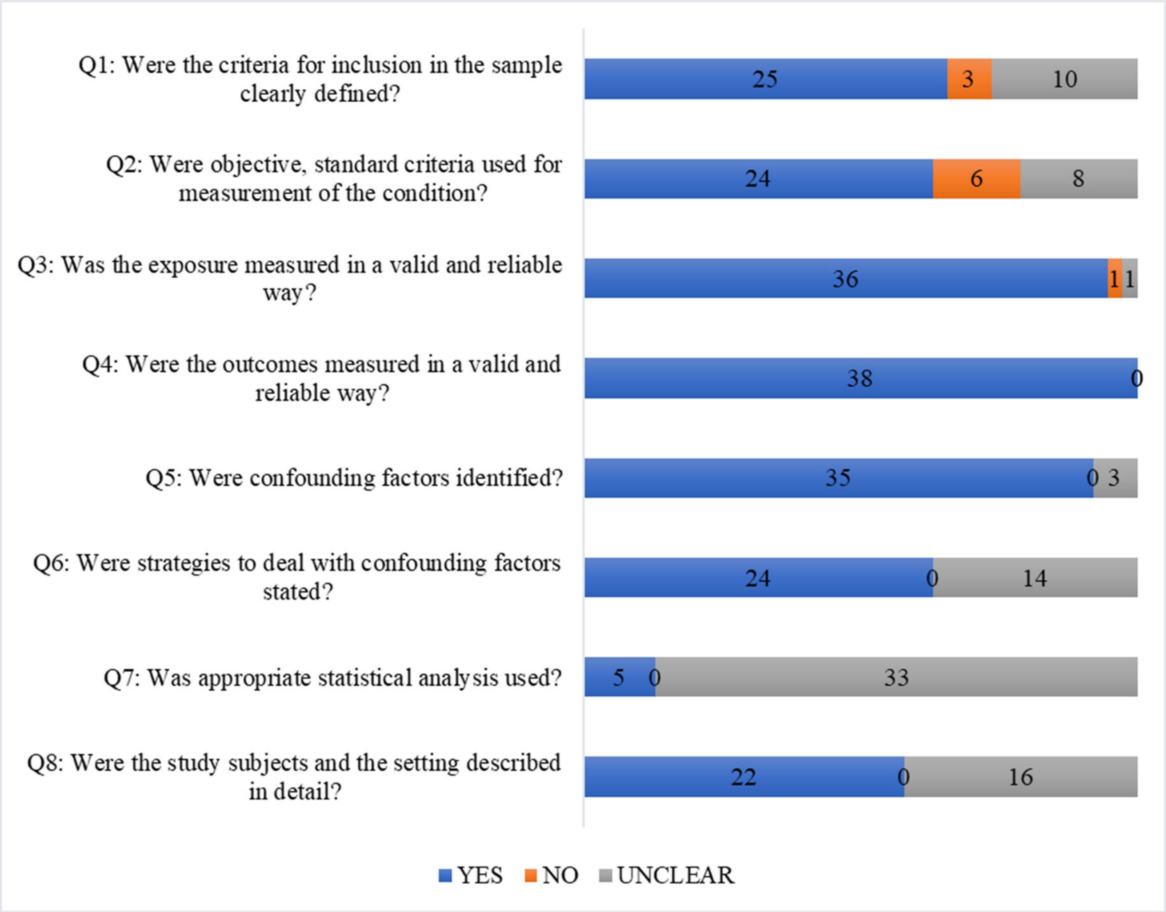

Notes: Summary of the number of studies (total N=38) rated as "YES", "NO", or "UNCLEAR" on each question (Q) of the JBI critical appraisal checklist. In Q2, the term "condition" refers to a Parkinson's Disease diagnosis. Aligned with the design of the included studies, in Q3, the term "exposure" refers to explanatory or independent variables whose association with an outcome is assessed, with an exclusive focus on cognition given the scope of the review. In Q4, decision-making was treated as the outcome. For Q7, the appraisal focused on statistical analyses investigating the association between cognition and decision-making, as these were the analyses relevant to the review's objectives. Special attention was given not only to whether appropriate statistical analyses had been applied but also to the robustness and methodological rigor of these analyses.
